# KMT2D‐mediated H3K4me1 recruits YBX1 to facilitate triple‐negative breast cancer progression through epigenetic activation of c‐Myc

**DOI:** 10.1002/ctm2.1753

**Published:** 2024-07-05

**Authors:** Bing Yao, Mengying Xing, Xiangwei Zeng, Ming Zhang, Que Zheng, Zhi Wang, Bo Peng, Shuang Qu, Lingyun Li, Yucui Jin, Haitao Li, Hongyan Yuan, Quan Zhao, Changyan Ma

**Affiliations:** ^1^ Department of Medical Genetics Nanjing Medical University Nanjing China; ^2^ Department of General Surgery, The Affiliated Taizhou People's Hospital of Nanjing Medical University, Taizhou School of Clinical Medicine Nanjing Medical University Taizhou China; ^3^ Jiangsu Key Laboratory of Xenotransplantation Nanjing Medical University Nanjing China; ^4^ The State Key Laboratory of Pharmaceutical Biotechnology School of Life Sciences Nanjing University Nanjing China; ^5^ MOE Key Laboratory of Protein Sciences Beijing Advanced Innovation Center for Structural Biology Beijing Frontier Research Center for Biological Structure Tsinghua‐Peking Joint Center for Life Sciences Department of Basic Medical Sciences School of Medicine Tsinghua University Beijing China; ^6^ School of Life Science and Technology China Pharmaceutical University Nanjing Jiangsu China; ^7^ Department of Oncology and Lombardi Comprehensive Cancer Center Georgetown University Medical Center Washington District of Columbia USA

**Keywords:** histone H3 lysine 4 mono‐methylation (H3K4me1), lysine methyltransferase 2D (KMT2D), triple‐negative breast cancer (TNBC), Y‐box‐binding protein 1 (YBX1)

## Abstract

**Background:**

Lysine methyltransferase 2D (KMT2D) mediates mono‐methylation of histone H3 lysine 4 (H3K4me1) in mammals. H3K4me1 mark is involved in establishing an active chromatin structure to promote gene transcription. However, the precise molecular mechanism underlying the KMT2D‐mediated H3K4me1 mark modulates gene expression in triple‐negative breast cancer (TNBC) progression is unresolved.

**Methods and Results:**

We recognized Y‐box‐binding protein 1 (YBX1) as a “reader” of the H3K4me1 mark, and a point mutation of YBX1 (E121A) disrupted this interaction. We found that KMT2D and YBX1 cooperatively promoted cell growth and metastasis of TNBC cells in vitro and in vivo. The expression levels of KMT2D and YBX1 were both upregulated in tumour tissues and correlated with poor prognosis for breast cancer patients. Combined analyses of ChIP‐seq and RNA‐seq data indicated that YBX1 was co‐localized with KMT2D‐mediated H3K4me1 in the promoter regions of c‐Myc and SENP1, thereby activating their expressions in TNBC cells. Moreover, we demonstrated that YBX1 activated the expressions of c‐Myc and SENP1 in a KMT2D‐dependent manner.

**Conclusion:**

Our results suggest that KMT2D‐mediated H3K4me1 recruits YBX1 to facilitate TNBC progression through epigenetic activation of c‐Myc and SENP1. These results together unveil a crucial interplay between histone mark and gene regulation in TNBC progression, thus providing novel insights into targeting the KMT2D‐H3K4me1‐YBX1 axis for TNBC treatment.

**Highlights:**

- YBX1 is a KMT2D‐mediated H3K4me1‐binding effector protein and mutation of YBX1 (E121A) disrupts its binding to H3K4me1.
- KMT2D and YBX1 cooperatively promote TNBC proliferation and metastasis by activating c‐Myc and SENP1 expression in vitro and in vivo.
- YBX1 is colocalized with H3K4me1 in the c‐Myc and SENP1 promoter regions in TNBC cells and increased YBX1 expression predicts a poor prognosis in breast cancer patients.

## Graphical abstract

- YBX1 is a “reader” of KMT2D‐mediated H3K4me1 mark.
- YBX1 and KMT2D activate the expression of SENP1 and c‐Myc.
- YBX1 is recruited by H3K4me1 mark to facilitate breast cancer progression.

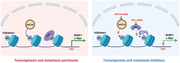

## 1 INTRODUCTION

Triple‐negative breast cancer (TNBC) is the most frequently diagnosed cancer and the leading contributor to cancer‐related deaths among women globally. The incidence and mortality rates of breast cancer are rapidly increasing,^1^ which can be attributed to various potential risk factors associated with economic development. These factors include changes in reproductive patterns, inadequate early screening methods, and limited access to treatment options.^2^, ^3^ In recent decades, significant efforts have been made to comprehend the pathogenesis of TNBC, particularly focusing on epigenetic events involved in oncogene activation and tumour suppressor gene inactivation.^4^, ^5^, ^6^ For instance, the C_p_G islands in the promoter region of tumour suppressor genes, such as p16^INK4A^, CCND2, CDH1, BRCA1, ER and RARβ2 exhibit hypermethylation, thus leading to gene expression silencing and facilitating tumour initiation and progression of TNBC.^7^, ^8^ Histone H3 methylation at lysine residues 4, 9, and 27 plays a key role in gene activation or inactivation in TNBC.^9^, ^10^, ^11^ Further research is needed to fully grasp the epigenetic changes in gene regulation and develop new treatment approaches for TNBC.^12^, ^13^

Lysine methyltransferase 2D (KMT2D), also called mixed‐lineage leukaemia 4 (MLL4), catalyzes mono‐methylation of histone H3 at lysine 4 (H3K4me1) in mammals, which participates in establishing an active chromatin structure and modulating specific gene transcription.^14^, ^15^, ^16^, ^17^, ^18^ KMT2D interacts with various transcription factors and coregulators to maintain an active chromatin state in different cell types.^15^, ^16^, ^17^, ^19^ Knockdown of KMT2D can impede the deposition of H3K4me1 on specific chromatin regions, thereby resulting in a repressive chromatin state and subsequent transcriptional inactivation.^20^ Functional investigations have demonstrated that KMT2D actively participates in the modulation of various cellular processes, such as cell growth, embryonic differentiation, and tumorigenesis, through H3K4me1‐related transcriptional regulation as well as the PI3K/Akt, Notch, and Wnt pathways.^21^, ^22^, ^23^, ^24^ Aberrant expression and mutation of KMT2D have been reported in multiple human malignancies, including lymphoma,^25^ ovarian,^26^ prostate,^27^ bladder,^28^ breast,^23^ and lung cancer.^29^

As a multifunctional transcription factor, YBX1 plays a role in gene regulation, RNA processing, and chromatin disruption.^30^, ^31^, ^32^ It is recognized as an oncogenic factor, contributing to tumour initiation and progression.^33^, ^34^, ^35^ Additionally, Kuwano et al.^36^ revealed that YBX1 activates ABCB1 gene expression by binding to its promoter region in cancer cells, thus facilitating malignant progression and drug resistance. Recently, Hartley et al.^37^ reported that protein arginine methyltransferase 5 (PRMT5)‐catalyzed YBX1 methylation was essential for NF‐κB activation and colorectal cancer progression. Numerous human cancers exhibit high expression of YBX1, such as bladder, ovarian and breast cancers, implicating YBX1 as a predictive marker for poor outcomes.^35^, ^38^, ^39^, ^40^, ^41^ Knockdown of YBX1 leads to suppression of genes in breast cancer cell cycle regulation, thus inhibiting tumour growth.^42^, ^43^, ^44^, ^45^

In this study, we discovered YBX1 as a potential “reader” of KMT2D‐catalyzed H3K4me1 mark. Moreover, we revealed that Glu121 was essential for YBX1 to recognize the H3K4me1 mark, and point mutation of YBX1 (YBX1‐E121A) could disrupt their interaction. Additionally, KMT2D and YBX1 cooperatively activated the expressions of c‐Myc and SENP1 to promote the growth, migration, and invasion of TNBC cells. We revealed a novel epigenetic regulatory axis, KMT2D‐H3K4me1‐YBX1, involved in oncogene activation, thereby providing new strategies for TNBC therapy.

## 2 MATERIALS AND METHODS

### 2.1 Cell culture

MDA‐MB‐231 and MDA‐MB‐468 cells were acquired from the Cell Bank, Chinese Academy of Sciences. Cell line authenticity was verified using short tandem repeat (STR) genotyping, and routine testing ensured the absence of mycoplasma contamination. RiboBio provided small interfering RNAs (siRNAs) targeting KMT2D and YBX1. Transfection of KMT2D or YBX1 siRNAs into cells was performed using Lipofectamine 3000 (Invitrogen). Target sequences for siRNAs were as follows:

KMT2D‐siRNA1: GCACCATCATTCGGAACGA;

KMT2D‐siRNA2: CCAGTACTTTCGCTTCGAA.

YBX1‐siRNA1: GGAACGGATATGGTTTCAT;

YBX1‐siRNA2: GGACGGCAATGAAGAAGAT.

### 2.2 Plasmid generation, recombinant protein purification

The recombinant plasmids pGEX and pET‐28a expressing GST‐YBX1 and His‐YBX1, respectively, were transformed into *E. coli* BL21 to generate two recombinant strains pGEX‐GST‐YBX1 and pET‐28a‐His‐YBX1. These strains were inoculated into LB medium and cultured with ampicillin or kanamycin, and isopropylthio‐β‐d‐galactoside at 16°C overnight. Post‐culturing, the cells were collected and sonicated in PBS. The resulting supernatants were incubated with either Glutathione Resin (GenScript Corp.) or High‐Affinity Ni‐Charged Resin (GenScript Corp.) beads for the purification of GST‐YBX1, His‐YBX1, and His‐YBX1‐E121A recombinant proteins. For the expression of KMT2D, SENP1 or c‐Myc, their respective coding sequence were integrated into the pcDNA3.1 vector to create the expression plasmids. The KMT2D_fusion_ mutant (Cys1523Ala, C1523A) was created with a site‐specific mutagenesis kit from Vazyme Biotech Corp.

### 2.3 Microscale thermophoresis assays

The Monolith NT protein labeling kit (Amine Reactive) was utilized to tag the fluorescent dye NT‐647‐NHS onto the cleansed YBX1 and YBX1‐E121A proteins. Proteins with labels were dissolved in a solution of PBS (pH 7.4) with 0.05% Tween‐20. Chemically synthesized H3, H3K4me1, and H3K4me3 peptides obtained from GenScript, within the range of 10 nM to 500 µM, were incubated with the labelled YBX1 or YBX1‐E121A proteins. After incubation, the mixture was moved to silica capillaries and thermophoresis was assessed using a NanoTemper Monolith NT.115 device from NanoTemper Technologies GmbH. Dissociation constant (Kd) values were determined using the law of mass action formula in NanoTemper Analysis 1.5.41 software. All microscale thermophoresis (MST) experiments were conducted at least three times.

### 2.4 Isothermal titration calorimetry assays

For the isothermal titration calorimetry (ITC) assay, a MicroCal Auto‐iTC200 Isothermal Titration Calorimetry system was employed. Primarily, chemically synthesized H3, H3K4me1, and H3K4me3 peptides along with the recombinant proteins underwent extensive dialysis using PBS. Subsequently, peptides and YBX1 were loaded into the ITC syringe at a concentration of 1 mM and 100 µM, respectively. Then, 2 µL of each of the H3, H3K4me1, and H3K4me3 peptides were injected into the ITC sample cell at 25°C. Analysis of the data was conducted with the assistance of Origin 8.0 software developed by OriginLab Corporation.

### 2.5 Molecular docking

We analyzed human YBX1 structure in complex with the H3K4me1 peptide to predict their binding interactions. We utilized the Glide module within the Schrödinger software suite (version: 2019‐3). The model of YBX1 (PDB:6KUG) was acquired from the Protein Data Bank and subsequently prepared in Maestro.

To prepare the protein, hydrogen atoms were added, water molecules and ions were removed, and the protein's structure was minimized using OPLS3e. The structure of the H3K4me1 peptide was generated using Builder in PyMOL, where it was designated as the ligand. The Van der Waals radii were adjusted to 0.8 and a partial charge cutoff of 0.15 was implemented for the docking parameters. The docking task utilized standard‐precision peptide without specifying YBX1 active site residues. Following the docking, poses from the result were thoroughly examined to assess the specificity of the interaction between YBX1 and H3K4me1 peptide.

### 2.6 Immunofluorescence

For immunofluorescence assay, cells were cultured in chamber slides and fixed with 4% paraformaldehyde solution at room temperature for 30 min. Following this, the cells were made permeable using 0.5% Triton X‐100. Nonspecific protein binding was then blocked with 5% BSA. The next step involved the addition of anti‐YBX1 and anti‐H3K4me1 antibodies to the cells, which were left to incubate overnight at 4°C. Following the wash, cells were subjected to a 1 h incubation at room temperature with the respective secondary antibody, after which they were stained with DAPI. Post‐staining, the slides were visualized using the confocal microscope.

### 2.7 Quantitative RT‐PCR (qRT‐PCR) and RNA sequencing assay

RNA was extracted using TRIzol reagent (Invitrogen Life Technologies). Subsequently, cDNAs were synthesized utilizing HiScript III 1st Strand cDNA Synthesis SuperMix (cat no. 11141; Yeasen) in accordance with the manufacturer's protocol. For qRT‐PCR, qPCR SYBR Green Master Mix (Yeasen) was employed in a CFX96 Touch Real‐Time PCR Detection System. The qRT‐PCR primer sequences can be seen in Table S7.

For RNA sequencing (RNA‐seq) assays, MDA‐MB‐231 cell RNA was isolated from NC, KMT2D‐KD and YBX1‐KD groups for library construction and sequencing. To enrich mRNA, the OligodT NEBNext Poly(A) mRNA Magnetic Isolation Module (NEB) was utilized, followed by fragmenting RNA to around 200 bp. Subsequent steps included first‐ and second‐strand cDNA synthesis, attaching adaptors, and enrichment according to the Illumina protocol. Purified library products were evaluated using Agilent 2200 Tape Station and Qubit (Thermo) Library sequencing was performed using the Illumina platform with end‐paired 150 bp. After collecting the data, clean reads were generated by eliminating sequences of poor quality, adapter impurities, and reads containing poly‐N. Alignment of clean reads to the human reference genome hg38 was accomplished utilizing HISAT2 under default parameters. The Illumina HiSeq 4000 system enabled the identification of aligned reads and the computation of FPKM values for individual genes. Gene expression analyses were conducted using specific criteria, such as a fold change greater than 2 and a *P*‐value less than 0.01. An additional understanding of the differentially expressed genes (DEGs) was revealed by analyzing Gene Ontology (GO) for ontological connections.

### 2.8 Cell viability, proliferation, and transwell assay

The in vitro viability of breast cancer cells was assessed using the cell counting kit‐8 (CCK‐8) assay kit. Briefly, 1 × 10^3^ cells were seeded in 96‐well plates following which CCK‐8 reagent (1/10; v/v) was added to each well. Following a 2 h incubation, the absorbance was determined at 450 nm. To assess cell proliferation, an EdU incorporation assay was performed utilizing the EdU Cell Proliferation Assay Kit (RiboBio) according to the protocol. For colony formation assays, 1 × 10^3^ cells were seeded in six‐well plates and cultured for 2 weeks. Afterwards, the cells were treated with ice‐methanol and stained using 0.1% crystal violet solution (Beyotime). For cell migration and invasion assays, 4 × 10^4^ and 8 × 10^4^ cells were seeded into the upper chambers of the transwell containing serum‐free medium (previously coated with Matrigel if used for invasion assays), while the lower chambers were filled with complete culture medium. After a 24 h incubation, the cells were subsequently stained using 0.1% crystal violet and quantified under a microscope.

### 2.9 Peptide pulldown

To identify H3K4me1‐binding proteins, peptide pulldown coupled with mass spectrometry was conducted. Specifically, a lysine‐methylated peptide consisting of 20 amino acids from H3, labelled with H3K4me1 and H3K4me3 at the C‐terminus using biotin (ART‐Kme0/1/3‐QTARKSTGGKAPRKQL), was synthesized by Genscript. For each peptide pulldown, 50 µL streptavidin magnetic beads (Beyotime; P2151) were mixed with 5 µg of peptide. Subsequently, the peptide‐bound beads underwent washing three times in binding buffer before being incubated with 500 µg of MDA‐MB‐231 cell lysate for 16 h at 4°C. The supernatant was discarded the following day and the streptavidin beads were washed three times for 10 min each in a wash buffer. The pulled‐down proteins were analyzed using mass spectrometry (Bioprofile).

### 2.10 Dot blot

Histone H3K4me1 and H3K4me3 peptides were diluted and directly spotted on nitrocellulose membranes. Following that, the membranes were blocked using 5% milk powder, then exposed to anti‐H3K4me1 (1:1000; Abcam; ab176877) and anti‐H3K4me3 (1:1000; Abcam; ab213224) antibodies for 16 h at 4°C. Next, the membranes were washed with 1×TBST in order to eliminate any antibodies that were not bound. Following washing, the membranes were exposed to secondary antibodies that were linked to HRP. Subsequently, chemiluminescence techniques were employed to detect signals on the membrane, and the level of binding was determined through image analysis to assess the specificity of the H3K4me1 and H3K4me3 antibodies.

### 2.11 Chromatin immunoprecipitation and re‐ChIP assays

For the chromatin immunoprecipitation (ChIP) assay, 5 × 10^6^ MDA‐MB‐231 cells were cross‐linked using 1% formaldehyde in an FBS‐free medium for 10 min at RT. To stop the crosslinking reaction, 0.12 M glycine solution was added for an additional 10 min. After washing twice with cold PBS, the cross‐linked chromatin was ultrasonically treated to obtain chromatin fragments of approximately 200−500 bp in length. 100 µg sonicated chromatin was immunoprecipitated with primary antibodies and salmon sperm DNA blocked protein A‐agarose beads overnight at 4°C. Following immunoprecipitation, the complexes were washed thrice with wash buffer supplemented with a protease inhibitor cocktail. For re‐ChIP experiments, eluted complexes from each sample of the primary ChIPs from the beads were treated with 50 µL of 10 mM dithiothreitol for 30 min at 37°C, followed by 3−4 times dilution in Dilution Buffer (1% SDS, 10 mM EDTA, 50 mM Tris‐HCl, pH 8.1). Following this, the re‐precipitation antibody was added and the ChIP assay procedure was repeated. The A‐agarose bead immunocomplexes were de‐crosslinked at 65°C, then exposed to proteinase K from Sigma, and finally precipitated with glycogen at a concentration of 20 mg/mL. Afterwards, the DNA that was immunoprecipitated was isolated from the complex of protein and DNA by utilizing a mixture of 25:24:1 phenol, chloroform, and isoamyl alcohol in a 1:1 ratio. Finally, the immunoprecipitated DNA was analyzed using qRT‐PCR, with the primer sequences detailed in Table S8.

### 2.12 ChIP‐sequencing

The immunoprecipitated DNA samples obtained from ChIP experiments using H3K4me1 and YBX1 antibodies underwent sequencing and analysis by Shanghai Jiayin Biotechnology Co., Ltd. Initial processing of the raw data, provided in fastq format, was conducted using in‐house Perl scripts to obtain clean data by eliminating reads containing adapters, ploy‐N, and low‐quality data. All subsequent analyses relied upon this high‐quality clean data. For aligning clean reads to the reference genome sequences, the bwa program was utilized. In the ChIP‐seq data analysis, average signal values for gene transcription start sites (TSS) and their flanking regions (1 kb upstream and downstream) were determined in both the NC and KMT2D‐KD or YBX1‐KD samples. For identifying genes with significant differences, a threshold was applied, necessitating a fold change exceeding two in average signal values. Specifically, the average ChIP‐seq signal value for each gene was computed, and the ratio between the KMT2D‐KD or YBX1‐KD and NC values was calculated.

### 2.13 Immunohistochemical staining

To examine the expressions of KMT2D, YBX1, SENP1, and c‐Myc using immunohistochemical (IHC) staining, we utilized a tissue microarray containing 140 breast cancer tissues and 77 adjacent normal tissues. IHC staining was performed according to standard methods, and antibodies were used against anti‐YBX1 (Abcam; ab239875), anti‐KMT2D (Abcam; ab224156), anti‐SENP1 (Abcam; ab108981), anti‐c‐Myc (Abcam; ab32072), or anti‐Ki‐67 (Abcam; ab15580). Finally, the sections were covered with glass coverslips (Beyotime; FCGF18) and viewed with a microscope (Nikon). The IHC score (H‐score) were evaluated by two independent pathologists. The quality of the tissue microarray was carefully assessed, and any data not meeting the criteria were excluded to ensure the accuracy of subsequent analysis. For instance, samples with section detachment were not included. Table S2 contains a list of the patients’ clinical characteristics.

### 2.14 Xenograft model and in vivo imaging

MDA‐MB‐231 cells were infected with pLKO.1 lentiviral control, KMT2D or YBX1 shRNA, following which puromycin‐resistant cells were selected. Subsequently, the stable cells were injected into mice for in vivo experiments. Six‐week‐old female nude mice were divided into three groups (five mice per group): (1) control (NC) group; (2) KMT2D knockdown (KMT2D‐KD) group; and (3) YBX1 knockdown (YBX1‐KD) group. Subcutaneous injection of 5 × 10^6^ stable NC, KMT2D‐KD, or YBX1‐KD MDA‐MB‐231 cells suspended in 100 µL of PBS containing 20% Matrigel was performed on BALB/c nude mice. Following 15 weeks, all mice were sacrificed and the xenograft tumours were excised.

MMTV‐PyMT mice were divided into three groups (five mice per group): (1) control (MMTV‐NC) group; (2) KMT2D knockdown (MMTV‐KMT2D‐KD) group; and (3) YBX1 knockdown (MMTV‐YBX1‐KD) group. Adeno‐associated virus (AAV) expressing murine KMT2D shRNA (GCACCATCATTCGCAATGA), YBX1 shRNA (GGAACGGATACGGTTTCAT) or the corresponding control shRNAs were administered to MMTV‐PyMT mice via the breast pad injection once weekly (1 × 10^8^ TU). The MMTV‐PyMT mice were sacrificed following 14 weeks. RNA and protein were isolated from tumour tissues, followed by an examination of lung metastatic nodules using H&E staining. For sacrificing mice, animals were killed by cervical dislocation after isoflurane anaesthesia. Animal experiments followed approved protocols from the Nanjing Medical University Laboratory Animal Center.

### 2.15 Statistical analysis

SPSS version 19.0 (IBM SPSS) and GraphPad Prism software (GraphPad Software, San Diego, CA) were used for statistical analysis of the data. Results are presented as mean ± standard deviation. Student's *t*‐test and ANOVA test were employed to calculate the statistical significance of each difference. Survival curves were generated by the Kaplan–Meier method and compared using the log‐rank test. Spearman correlation analysis was used to assess relationships between groups. *p* < 0.05 was statistically significant.

## 3 RESULTS

### 3.1 YBX1 is an H3K4me1‐binding protein

**FIGURE 1:**
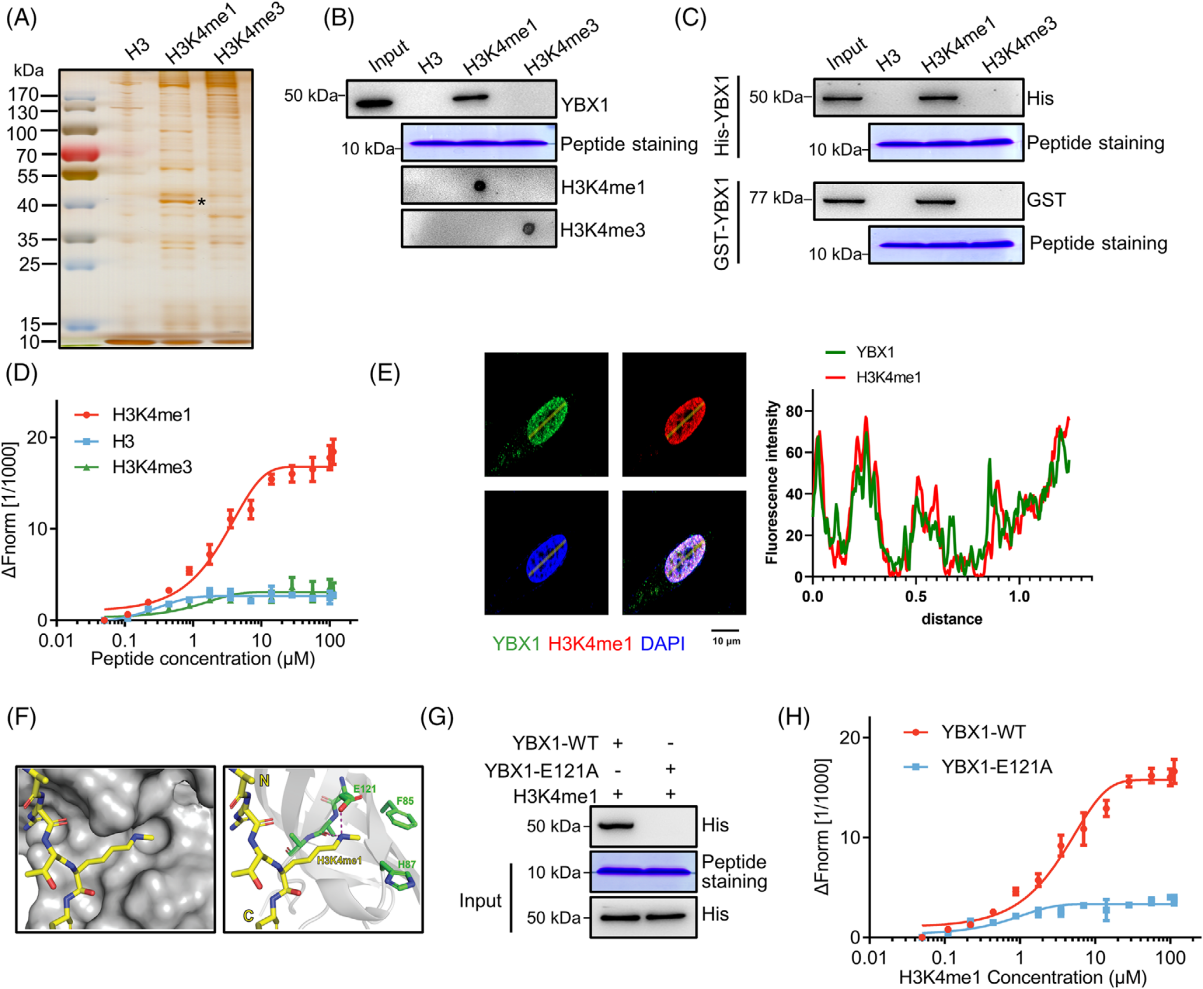
YBX1 is an H3K4me1‐binding protein and YBX1‐E121 is necessary for its binding. (A) Peptide pull‐down assay using H3, H3K4me1 and H3K4me3 peptides incubated with MDA‐MB‐231 cell lysate. (B) Peptide pull‐down assay to detect the interactions between H3, H3K4me1 and H3K4me3 peptides and YBX1. Coomassie Blue staining representing H3, H3K4me1 and H3K4me3 peptide. The Dot blot experiment was used to validate the synthesized H3K4me1 and H3K4me3 peptides. (C) Peptide pull‐down assay to detect the interaction between H3, H3K4me1 and H3K4me3 peptides and purified recombinant His‐ or GST‐YBX1. Coomassie Blue staining representing H3, H3K4me1 and H3K4me3 peptide. (D) MST assay was performed to detect the interactions between His‐YBX1 and H3, H3K4me1 or H3K4me3 peptides. (E) Colocalization analysis of YBX1 (green) and H3K4me1 (red) in MDA‐MB‐231 cells. Scale bar = 10 µm. (F) The overall structure of the YBX1‐H3K4me1 complex. Grey, CSD domain of YBX1; green, Glu121 (E121), Phe85 (F85) and His87 (H87) of CSD domain; yellow, histone H3 tail peptide. (G) Peptide pull‐down experiments were performed with H3K4me1 peptide and purified recombinant His‐YBX1 and His‐YBX1‐E121A mutant expressed in *E. coli*. (H) MST assay to detect the interactions between H3K4me1 peptide and YBX1‐WT or YBX1‐E121A recombinant protein.

The role of H3K4me1 in regulating the chromatin environment and assembly of transcription machinery has been firmly established, as it regulates the recruitment of effector proteins to gene promoter regions.^46^ To identify “reader” proteins that recognize H3K4me1, we incubated MDA‐MB‐231 cell lysate with methylated and unmethylated H3 peptides, followed by separation with SDS‐PAGE gel and staining with Silver SafeStain (Figure 1A). The Mass spectrometry analysis revealed a significant enrichment of YBX1 in the precipitates of the H3K4me1 peptide when compared with H3 and H3K4me3 peptides (Table S1). Peptide pull‐down assays further confirmed the selective interaction between YBX1 and H3K4me1 peptide (Figure 1B). Dot blot analyses successfully demonstrated the high specificity of the H3K4me1 and H3K4me3 antibodies towards their corresponding modifications (Figure 1B). Moreover, in vitro pull‐down assays using purified recombinant YBX1 demonstrated direct interactions between YBX1 and H3K4me1 peptide (Figure 1C, Figure S1A, B). The result of MST assay revealed that His‐YBX1 protein bound to the H3K4me1 peptide while showing no discernible binding affinity towards either H3 or H3K4me3 peptides (Figure 1D). Similar results were obtained from the ITC assay (Figure S1C). In MDA‐MB‐231 cells, immunofluorescence staining showed co‐localization of YBX1 and H3K4me1 marks in the nucleus (Figure 1E). The molecular docking analysis revealed that H3K4me1 can snugly fit into a surface cavity formed by the hydrophobic side chains of Glu121 (E121), Phe85 (F85), and His87 (H87) residues in YBX1 (Figure 1F). Interestingly, the main‐chain carbonyl group of E121 in YBX1 was found to be in close proximity to the amino group of H3K4, allowing for potential hydrogen‐bonding interactions that may contribute to its preferential binding to H3K4me1 (Figure 1F). Moreover, the monomethyl group of H3K4 peptide was enclosed within a preformed cavity of precise dimensions, effectively excluding the binding of dimethylated or trimethylated H3K4 (H3K4me2 or H3K4me3). In order to further validate the result of virtual docking, we introduced point mutations of YBX1 at E121 (E121A), Phe85 (F85A), and His87 (H87A) within the binding pocket and found that only E121A mutation of YBX1 entirely abolished its interaction with H3K4me1 mark (Figure 1G, Figure S1D). Consistently, the MST assay showed that E121A mutation significantly attenuated the binding affinity of YBX1 to H3K4me1 (Kd = 82.6 ± 4.1 µM; Figure 1H). Based on these findings, YBX1 appears to be an H3K4me1 reader, with residue E121 playing a vital role in mediating this interaction.

### 3.2 KMT2D and YBX1 promote TNBC cell proliferation and migration

**FIGURE 2:**
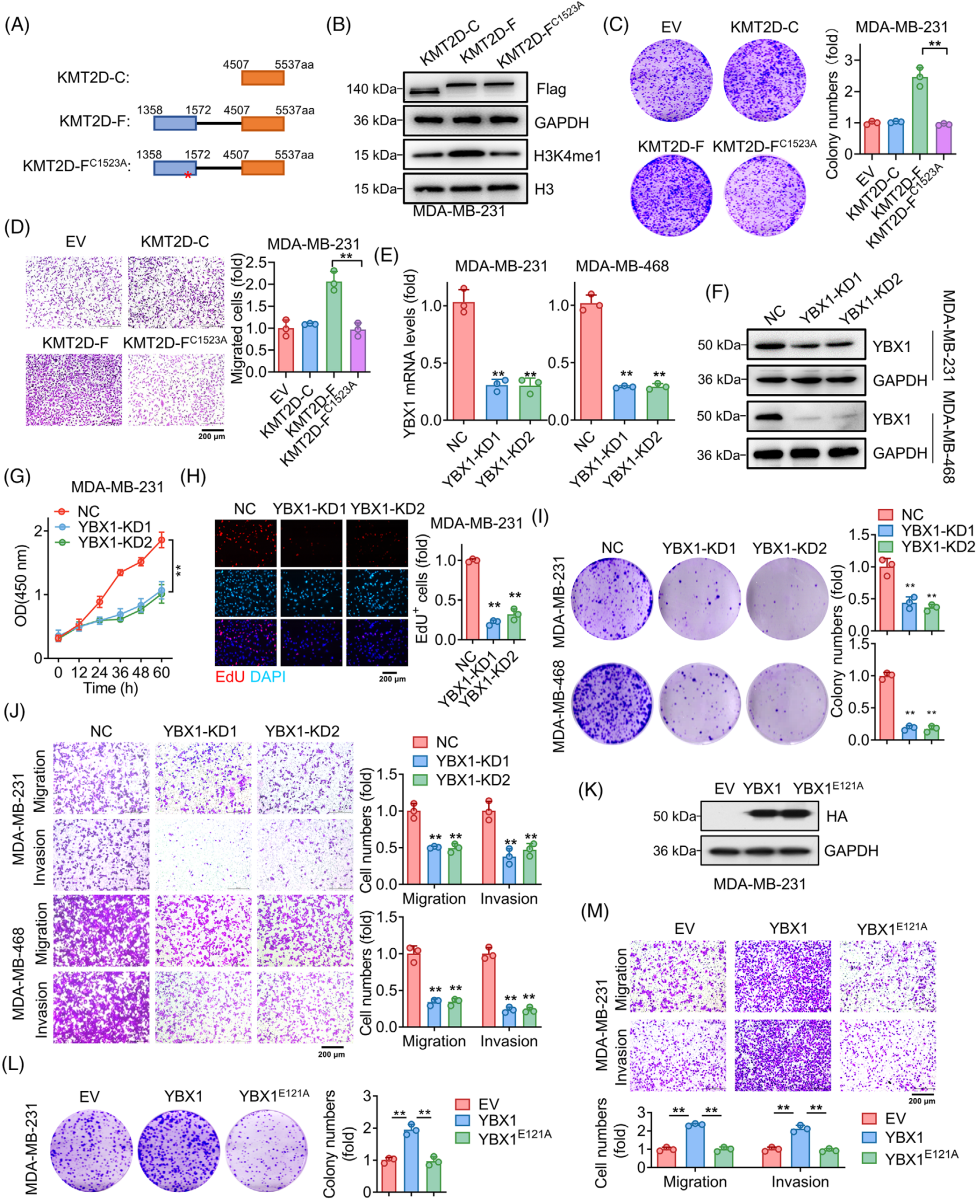
KMT2D and YBX1 promote TNBC cell proliferation and migration. (A) Schematic representation of KMT2D‐C, KMT2D‐F, and KMT2D‐F^C1523A^. (B) Western blot analysis of H3K4me1 in KMT2D‐C, KMT2D‐F, and KMT2D‐F^C1523A^‐overexpressed MDA‐MB‐231 cells. (C, D) Colony‐formation (C) and migration (D) assays were performed to examine cell growth and migration of empty vector (EV), KMT2D‐C, KMT2D‐F, and KMT2D‐F^C1523A^‐overexpressed MDA‐MB‐231 cells. (E, F) qRT‐PCR (E) and Western blot (F) analysis of YBX1 mRNA and protein levels in MDA‐MB‐231 and MDA‐MB‐468 cells transfected with negative control (NC) or YBX1 siRNAs. (G, H) CCK‐8 (G) and EdU (H) assays were performed to examine the proliferation of control (NC) and YBX1‐knockdown (YBX1‐KD) in MDA‐MB‐231 cells. (I, J) Colony formation (I), migration and invasion (J) assay of NC and YBX1‐KD in MDA‐MB‐231 and MDA‐MB‐468 cells. (K) Western blot analysis of HA‐tag protein levels in MDA‐MB‐231 cells transfected with empty vector (EV), YBX1‐WT or YBX1‐E121A constructs. (L, M) Colony formation (K), migration and invasion (L) assay of MDA‐MB‐231 cells transfected with empty vector (EV), YBX1‐WT or YBX1‐E121A constructs. Data were shown as mean ± SD. **p < 0.01.

Given the fact that H3K4me1 is mainly catalyzed by KMT2D in mammals,^15^ we next explored the roles of KMT2D in breast cancer cells. Using two TNBC cell lines, we silenced KMT2D (Figure S2A, B) and found significant reductions in cell growth (Figure S2C–E), migration, and invasion (Figure S2F) in KMT2D‐knockdown cells, which aligns with previously reported findings by Kim et al.^47^ To investigate the dependence of KMT2D's effect on its methyltransferase activity, we generated a series of truncated KMT2D‐expressing vectors based on a previously published protocol.^48^ As illustrated in Figure 2A, KMT2D‐C contains C‐terminus 4507 to 5537 amino acids of KMT2D, KMT2D‐F is a fusion protein containing PHD domains and C‐terminus of KMT2D and KMT2D‐F^C1523A^ is a loss‐of‐function mutant of KMT2D‐F. Consistent with previous findings, overexpression of KMT2D‐F significantly enhanced H3K4me1 levels in MDA‐MB‐231 cells, whereas such effect was not observed with either KMT2D‐C or KMT2D‐F^C1523A^ (Figure 2B). In consistence, overexpression of KMT2D‐F promoted cell growth and migration compared with KMT2D‐C‐ and KMT2D‐F^C1523A^‐overexpressing MDA‐MB‐231 cells (Figure 2C–D). Based on these results, KMT2D facilitates cell growth and migration in TNBC cells in a methyltransferase activity‐dependent manner.

As we found that YBX1 was a potential “reader” of KMT2D‐mediated H3K4me1, we next investigated theroles of YBX1 in TNBC cells. Upon silencing YBX1 (Figure 2E, F), a significant inhibitory effect was observed on the proliferation of TNBC cells (Figure 2G–I). Additionally, YBX1 knockdown reduced TNBC cell migration and invasion in transwell assays (Figure 2J). Conversely, MDA‐MB‐231 cells overexpressing YBX1 and not YBX1‐E121A exhibited enhanced migration and proliferation (Figure 2K–M). It appears that KMT2D and YBX1 participation in influencing H3K4me1 mark writing and reading is essential for the proliferation, migration, and invasion of TNBC cells.

### 3.3 c‐Myc and SENP1 are target genes of KMT2D and YBX1 in TNBC cells

**FIGURE 3:**
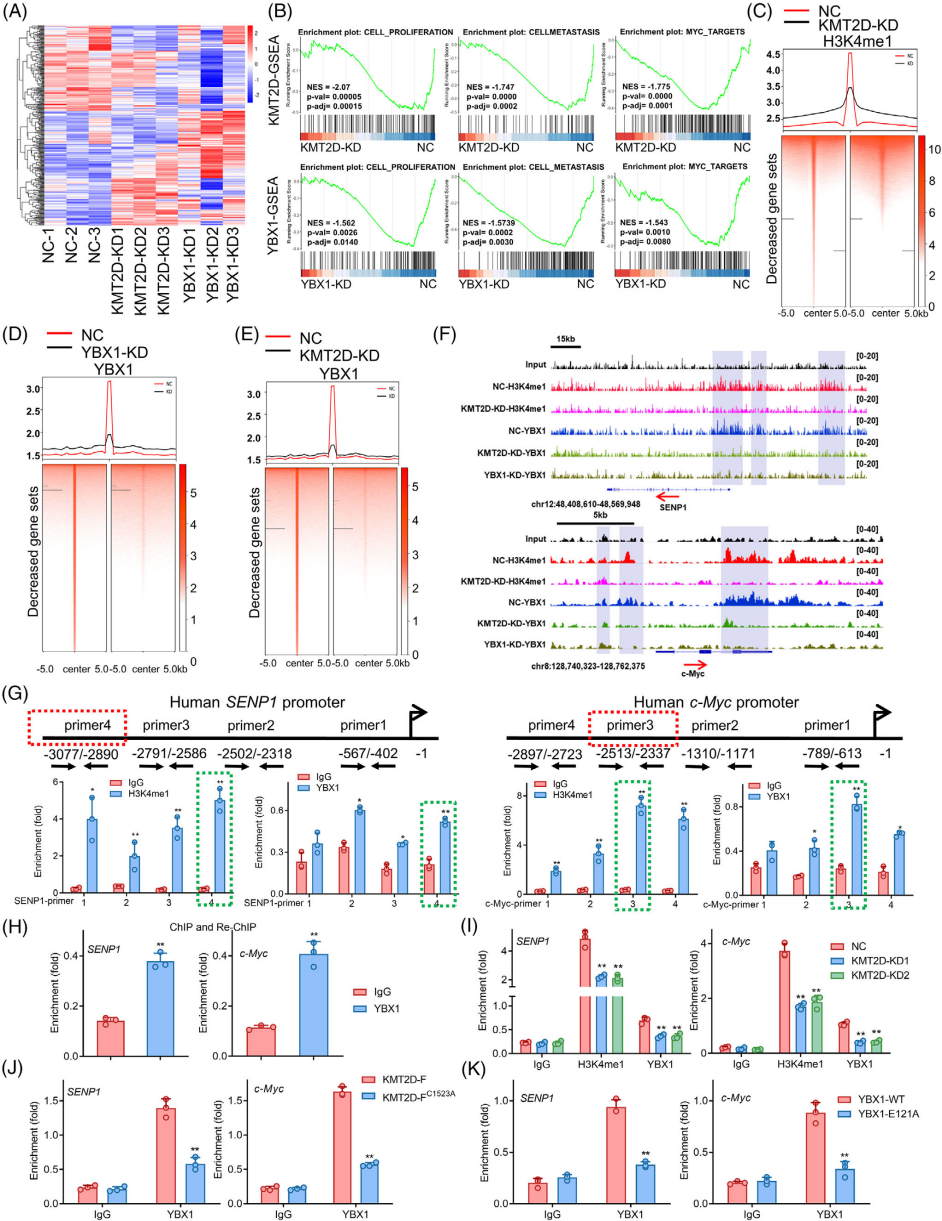
c‐Myc and SENP1 are target genes of KMT2D and YBX1 in TNBC cells. (A) Differentially expressed genes (DEGs) after KMT2D (KMT2D‐KD1, KMT2D‐KD2 and KMT2D‐KD3) or YBX1 knockdown (YBX1‐KD1, YBX1‐KD2 and YBX1‐KD3), relative to negative control (NC‐1, NC‐2, and NC‐3) in MDA‐MB‐231 cells. (B) Gene set enrichment analysis (GSEA) of cell proliferation, cell metastasis and Myc target genes in KMT2D‐knockdown or YBX1‐knockdown MDA‐MB‐231 cells. (C) Heatmap of input normalized H3K4me1 ChIP signal in NC, KMT2D‐KD over 3749 distal H3K4me1 regions with decreased signals in KMT2D‐KD, with regions sorted by strength of H3K4me1 signal. (D) Heatmap of input normalized YBX1 ChIP signal in NC and YBX1‐KD over 3638 distal YBX1 regions with decreased signals in YBX1‐KD, with regions sorted by strength of YBX1 signal. (E) Heatmap of input normalized YBX1 ChIP signal in NC and KMT2D‐KD over 3362 distal YBX1 regions with decreased signals in KMT2D‐KD, with regions sorted by strength of YBX1 signal. (F) Browser shots of ChIP‐seq signal for YBX1 and H3K4me1 at the SENP1 and c‐Myc locus in control, KMT2D‐ (KMT2D‐KD) or YBX1‐knockdown (YBX1‐KD) MDA‐MB‐231 cells. (G) ChIP assay showing the binding of H3K4me1 and YBX1 to c‐Myc and SENP1 promoters. (H) ChIP‐reChIP analysis using anti‐H3K4me1 as the first antibody and anti‐YBX1 as the second antibody. Relative enrichments of H3K4me1 and YBX1 on the promoters of c‐Myc and SENP1 were determined by qPCR analysis. (I) ChIP analysis of H3K4me1 and YBX1 binding to promoters of c‐Myc and SENP1 in control (NC) and KMT2D‐knockdown (KMT2D‐KD1 and KMT2D‐KD2) MDA‐MB‐231 cells. (J) ChIP analysis of YBX1 on the promoters of c‐Myc and SENP1 in KMT2D‐F^C1523A^‐overexpressed cells or control (KMT2D‐F) cells. (K) ChIP analysis of YBX1 on the promoters of c‐Myc and SENP1 in YBX1‐E121A cells or control (YBX1‐WT) cells. Data were shown as mean ± SD. *p < 0.05, **p < 0.01.

Subsequently, we investigated the target genes of KMT2D and YBX1 in TNBC cells. To assess the global transcriptional alterations in KMT2D‐ or YBX1‐knockdown MDA‐MB‐231 cells, we conducted an RNA‐sequencing analysis (Figure 3A). As a result of gene set enrichment analysis (GSEA), significant enrichments were found for proliferation, metastasis, and Myc target genes, which are hallmarks of many malignancies (Figure 3B). Notably, the gene ontology (GO) analysis revealed that KMT2D and YBX1‐silenced DEGs were highly enriched in processes including Wnt signalling, cellular proliferation, and migration (Figure S3A). 218 genes were increased and 266 genes were decreased in MDA‐MB‐231 cells with KMT2D knockdown, while 2581 genes were elevated and 1070 genes were reduced in cells with YBX1 knockdown (Figure S3B). Among the downregulated genes, 36 genes were found to be common in both KMT2D‐ and YBX1‐knockdown cells (Table S3). Moreover, ChIP‐seq experiments were conducted to examine the global distribution of the H3K4me1 modification and the YBX1 protein. The ChIP‐seq analysis revealed that 22.22% of H3K4me1 peaks and 37.92% of YBX1 peaks were distributed within the promoter region of the genome, respectively (Figure S3C). Importantly, our findings indicated that KMT2D‐knockdown resulted in a significant reduction of H3K4me1 and YBX1 signals at the TSSs (Figure 3C–E). Moreover, our ChIP‐seq data identified 329 overlapping genes involving the H3K4me1 mark and YBX1 in the genome (Figure S3D). The combined examination of ChIP‐seq and RNA‐seq revealed c‐Myc and small ubiquitin‐like modifier (SUMO)‐specific protease 1 (SENP1), as key genes influenced by both KMT2D and YBX1, with SENP1 being an important deSUMOylating enzyme that enhances c‐Myc stability and function (Figure S3E). Consistent with this, ChIP‐seq data demonstrated the enrichment of YBX1 and H3K4me1 on the promoters of SENP1 and c‐Myc. Additionally, knockdown of KMT2D notably attenuated their binding affinity to these regions (Figure 3F).

Next, we conducted ChIP assays to confirm the enrichment of H3K4me1 and YBX1 on the promoters of *SENP1* and *c‐Myc*. The results showed notable occupancy of H3K4me1 and YBX1 at the promoter regions of *SENP1* and *c‐Myc* genes (Figure 3G, Figure S3F). Furthermore, utilizing a ChIP‐reChIP assay, we demonstrated an interaction between H3K4me1 and YBX1 on the promoters of *c‐Myc* and *SENP1* by re‐immunoprecipitating chromatin initially immunoprecipitated with H3K4me1 antibody using an antibody specific to YBX1 (Figure 3H). Moreover, KMT2D‐knockdown resulted in a substantial decrease of H3K4me1 and YBX1 occupancy on the promoters of *SENP1* and *c‐Myc* (Figure 3I). Similarly, due to a dominant‐negative effect, overexpression of KMT2D‐F^C1523A^ decreased YBX1 binding on the *c‐Myc* and *SENP1* promoters compared with that in KMT2D‐F‐overexpressed MDA‐MB‐231 cells (Figure 3J). Additionally, the YBX1^E121A^ mutation also led to reduced binding of YBX1 on *c‐Myc* and *SENP1* promoters (Figure 3K). These findings together suggest that KMT2D‐mediated H3K4me1 recruits YBX1 to the promoters of *SENP1* and *c‐Myc* in MDA‐MB‐231 cells.

### 3.4 KMT2D and YBX1 promote transcriptional expression of *c‐Myc* and *SENP1*

**FIGURE 4:**
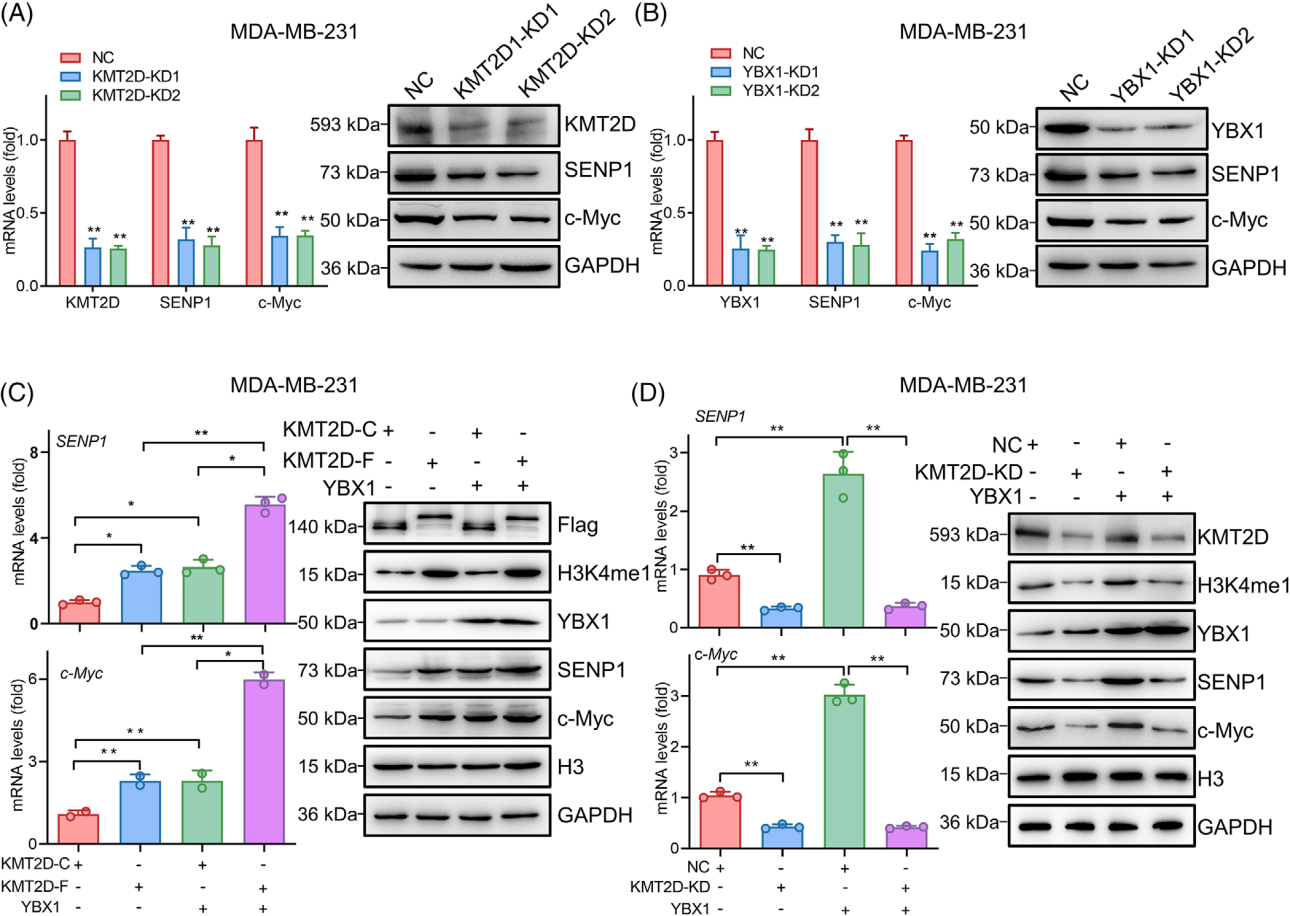
KMT2D and YBX1 promote the transcriptional expression of c‐Myc and SENP1. (A‐B) qRT‐PCR and Western blot analysis of the mRNA and protein levels of c‐Myc and SENP1 in KMT2D‐ (A) or YBX1‐knockdown (B) MDA‐MB‐231 cells, relative to negative control (NC). (C) mRNA and protein expression levels of c‐Myc and SENP1 in MDA‐MB‐231 cells transfected with KMT2D‐C, KMT2D‐F, or YBX1 expression vectors. (D) mRNA and protein expression levels of c‐Myc and SENP1 in MDA‐MB‐231 cells transfected with negative control (NC), KMT2D siRNA (KMT2D‐KD) or YBX1 expression vector. Data were shown as mean ± SD. *p < 0.05, **p < 0.01.

Subsequently, we explored the roles of KMT2D and YBX1 on the expressions of c‐Myc and SENP1 in TNBC cells. Knockdown of KMT2D and YBX1 effectively suppressed the expressions of SENP1 and c‐Myc in TNBC cells, respectively (Figure 4A‐B, Figure S3G, H). Considering the regulatory effect of either KMT2D or YBX1 on the expressions of c‐Myc and SENP1 in TNBC cells, we aimed to investigate whether a cooperative regulation exists between YBX1 and KMT2D regarding their influence on c‐Myc and SENP1 expression levels. Our findings revealed that independent activation of mRNA and protein expression for c‐Myc as well as SENP1 was observed upon overexpression of either KMT2D‐F or YBX1, while co‐expression of both factors exhibited a synergistic effect (Figure 4C). In contrast, KMT2D knockdown almost completely abolished the promotion effect of YBX1 on the expressions of SENP1 and c‐Myc (Figure 4D). These data together suggest a highly synergistic transcriptional activation mechanism between KMT2D and YBX1 for the expressions of c‐Myc and SENP1.

### 3.5 KMT2D and YBX1 cooperatively promote TNBC cell growth and migration

**FIGURE 5:**
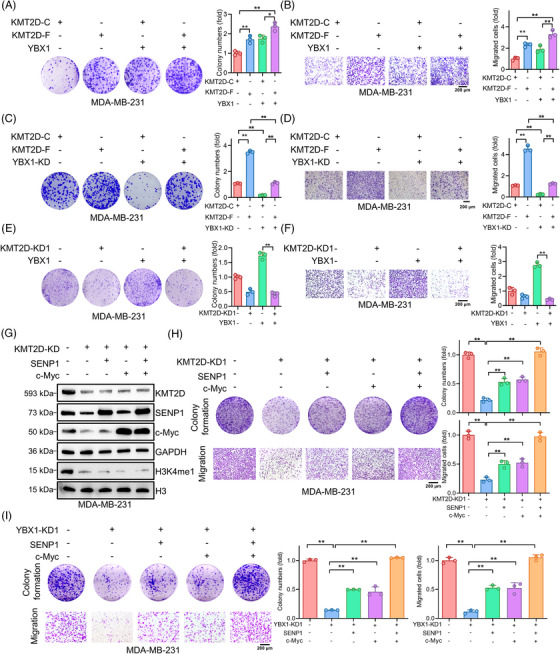
KMT2D and YBX1 cooperatively promote TNBC cell proliferation and migration. (A, B) Colony formation (A) and migration (B) assays of MDA‐MB‐231 cells transfected with KMT2D‐C, KMT2D‐F, KMT2D‐C + YBX1 and KMT2D‐F + YBX1. (C, D) Colony formation (C) and migration (D) assays of MDA‐MB‐231 cells transfected with KMT2D‐C, KMT2D‐F, KMT2D‐C + YBX1‐KD and KMT2D‐F + YBX1‐KD. (E, F) Colony formation (E) and migration (F) assays of MDA‐MB‐231 cells transfected with NC, KMT2D‐KD1, YBX1, KMT2D‐KD1 + YBX1. (G) Western blot analysis of c‐Myc and SENP1 protein expression levels in MDA‐MB‐231 cells transfected with NC, KMT2D‐KD, SENP1 or c‐Myc. (H) Colony formation and migration assays of MDA‐MB‐231 cells transfected with NC, KMT2D‐KD1, SENP1 or c‐Myc. (I) Colony formation and migration assays of MDA‐MB‐231 cells transfected with NC, YBX1‐KD1, SENP1 or c‐Myc.

We next explored whether there existed an addictive impact of KMT2D and YBX1 on the growth and migration of TNBC cells. Co‐overexpression of both KMT2D‐F and YBX1 enhanced proliferation and migration more than overexpression of either protein alone (Figure 5A, B). Compared with KMT2D‐C‐overexpressing MDA‐MB‐231 cells, KMT2D‐F‐overexpression significantly increased proliferation, migration, and invasion, with YBX1 knockdown attenuating these effects (Figure 5C, D). Conversely, KMT2D knockdown significantly attenuated the proliferative and migratory (Figure 5E, F; Figure S4A, B) effects induced by YBX1, indicating a KMT2D‐dependent role of YBX1. Additionally, reintroduction of SENP1 or c‐Myc (Figure 5G) significantly rescued the proliferation and migration of KMT2D‐ (Figure 5H, Figure S4C) and YBX1‐knockdown cells (Figure 5I, Figure S4D). Based on these results, YBX1 and KMT2D cooperatively facilitate the growth and migration by activating c‐Myc and SENP1 expressions in TNBC cells.

### 3.6 Knockdown of KMT2D or YBX1 attenuates breast tumour growth and metastasis

**FIGURE 6:**
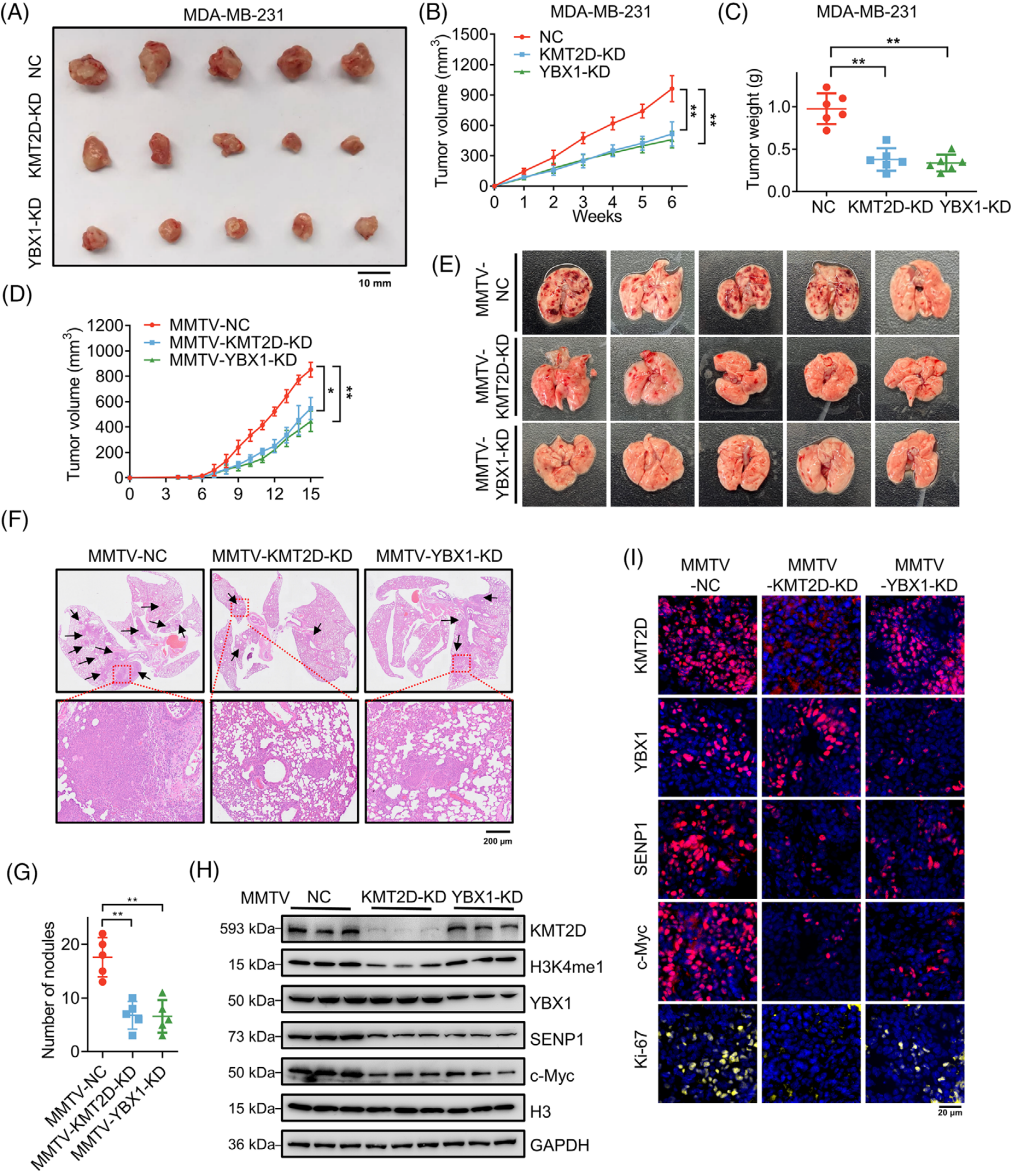
Knockdown of KMT2D or YBX1 attenuates breast tumor growth and metastasis. (A) Tumour tissues of NC, KMT2D‐knockdown (KMT2D‐KD) and YBX1‐knockdown (YBX1‐KD) MDA‐MB‐231 xenografts. (B) Tumour growth curves of NC, KMT2D‐knockdown (KMT2D‐KD) and YBX1‐knockdown (YBX1‐KD) MDA‐MB‐231 xenografts. (C) Tumour weights of NC, KMT2D‐knockdown (KMT2D‐KD) and YBX1‐knockdown (YBX1‐KD) MDA‐MB‐231 xenografts. (D) The mammary tumour growth curves (the size of the first tumour observed in each mouse) of NC, KMT2D‐KD and YBX1‐KD AAV‐treated MMTV‐PyMT transgenic mice. (E) Lung metastatic nodules in NC, KMT2D‐KD and YBX1‐KD AAV‐treated MMTV‐PyMT transgenic mice. (F) Representative H&E staining images of lung metastatic nodules from NC, KMT2D‐KD and YBX1‐KD AAV‐treated MMTV‐PyMT transgenic mice, and black arrows indicate metastatic nodules. Scale bar = 200 µm. (G) Statistical results of lung metastatic nodules from NC, KMT2D‐KD and YBX1‐KD AAV‐treated MMTV‐PyMT transgenic mice. (H) Western blot analysis of the protein levels of KMT2D, H3K4me1, YBX1, SENP1, and c‐Myc in mammary tumour tissues from NC, KMT2D‐KD and YBX1‐KD AAV‐treated MMTV‐PyMT transgenic mice. (I) Immunofluorescence to examine the expression of KMT2D, YBX1, SENP1, c‐Myc, and Ki‐67 in mammary tumour tissues from NC, KMT2D‐KD and YBX1‐KD AAV‐treated MMTV‐PyMT transgenic mice.

Next, we investigated the roles of KMT2D and YBX1 using mouse models of breast cancer. In mouse xenograft model, knockdown of either KMT2D or YBX1 inhibited tumour growth (Figure 6A, B). The weights of MDA‐MB‐231 xenograft tumours significantly decreased when KMT2D or YBX1 were knocked down (Figure 6C). As well, xenograft tumours derived from KMT2D‐ and YBX1‐knockdown cells expressed significantly lower levels of c‐Myc and SENP1 (Figure S5A, B). Additionally, tumour tissues from the KMT2D‐ or YBX1‐knockdown groups showed reduced expression of Ki‐67, a well‐established marker of cell proliferation (Figure S5B).

The use of AAV vectors to knockdown KMT2D and YBX1 in the mammary glands of MMTV‐PyMT transgenic (MMTV‐KMT2D‐KD or MMTV‐YBX1‐KD) mice enabled us to test their therapeutic potential in breast cancer. The efficiency of AAV infection was confirmed by detecting GFP fluorescence. Remarkably, both AAV‐KMT2D‐KD and AAV‐YBX1‐KD mice exhibited reduced tumour growth and lung metastasis (Figure 6D, E). Additionally, H&E staining and microscopic examination revealed that both MMTV‐KMT2D‐KD and MMTV‐YBX1‐KD mice showed substantially fewer lung metastatic nodules when compared with control mice (Figure 6F, G). Consistently, mammary tumour tissues from both MMTV‐KMT2D‐KD and MMTV‐YBX1‐KD mice showed decreased expression levels of SENP1, c‐Myc and Ki67 (Figure 6H, I, Figure S5C). Collectively, these findings suggest that knockdown of KMT2D and YBX1 impairs breast cancer growth and metastasis.

### 3.7 Elevated KMT2D and YBX1 expression predicts an unfavorable prognosis of breast cancer patients

**FIGURE 7:**
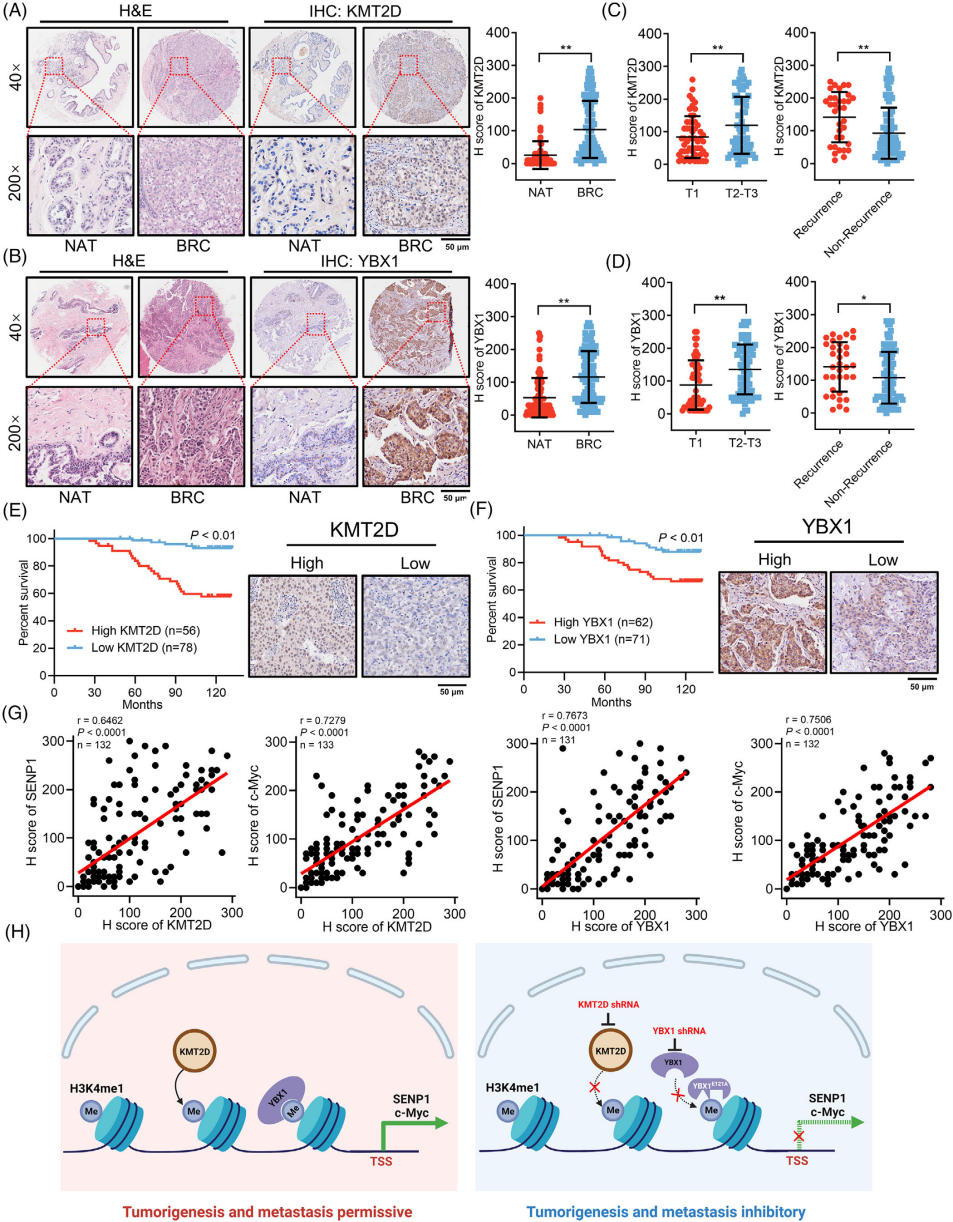
Elevated KMT2D and YBX1 expression positively correlates with c‐Myc and SENP1 expression and predicts an unfavourable prognosis in patients with breast cancer. (A) Representative HE staining and IHC of KMT2D in adjacent normal (NAT) and breast cancer (BRC) tissues. Scale bar = 50 µm. The right panel shows the IHC score of KMT2D in adjacent normal and breast cancer tissues. (B) Representative HE staining and IHC of YBX1 in NAT and RBC tissues. The right panel shows the IHC score of YBX1 in adjacent normal and breast cancer tissues. Scale bar = 50 µm. (C) Correlation of KMT2D expression with tumour stages and recurrence. (D) Correlation of YBX1 expression with tumour stages and recurrence. (E) Kaplan‐Meier survival analysis of 134 breast cancer patients, stratified by KMT2D expression (log‐rank test, *p* < 0.01). (F) Kaplan–Meier plot survival analysis of 133 breast cancer patients, stratified by YBX1 expression (log‐rank test, *p* < 0.01). (G) Pearson correlation analysis of H scores of SENP1 and KMT2D (*r* = 0.6462, *p *< 0.0001), c‐Myc and KMT2D (*r* =0.7279, *p* < 0.0001), SENP1 and YBX1 (*r* =0.7673, *p* < 0.0001), c‐Myc and YBX1 (*r* =0.7506, *p* < 0.0001) in human breast cancer tissues. (H) A schematic model illustrating the roles of KMT2D and YBX1 in aggravating breast cancer progression through epigenetically activating c‐Myc and SENP1 expression.

In order to assess the clinical significance of KMT2D and YBX1 in breast cancer, we used a tissue microarray containing 140 breast cancer tissues and 77 adjacent healthy tissues. The expression of KMT2D and YBX1 was upregulated in tumour tissues when compared with adjacent tissues, as shown in Figure 7A, B. In addition, KMT2D and YBX1 were significantly overexpressed in the tumour tissues of patients with advanced TNM stage and recurrence (Figure 7C, D and Table S2). The Kaplan–Meier survival analysis showed a significant association between elevated expressions of KMT2D and YBX1 and poor survival of breast cancer patients within this cohort (Figure 7E, F). On this microarray, we also examined c‐Myc and SENP1. According to IHC staining, both c‐Myc and SENP1 were upregulated in tumour tissues compared with adjacent healthy tissues (Figure S6A, B) and associated with short survival for breast cancer patients (Figure S6C, D). Furthermore, the levels of SENP1 and c‐Myc showed a positive correlation with KMT2D and YBX1 in the breast cancer tissues analyzed (Figure 7G) as well as 17 TNBC tissues in this cohort (Figure S6E). Collectively, these findings indicate that the levels of KMT2D and YBX1 are associated with the levels of c‐Myc and SENP1, suggesting a poor prognosis for individuals with breast cancer. As a result, the KMT2D‐H3K4me1‐YBX1 axis may facilitate breast cancer progression by positively modulating the expressions of c‐Myc and SENP1 (Figure 7H).

## 4 DISCUSSION

Genome‐wide analysis of histone modifications has demonstrated that H3K4me1 is preferentially deposited at a large set of active and primed enhancers.^15^, ^49^ The presence of H3K4me1 blocks the binding of H3K4me3‐associated factors and demarcates enhancer boundaries, thereby restricting the recruitment of multiple chromatin regulators.^50^, ^51^ However, whether and how this histone mark modulates enhancer‐independent transcription remains elusive. KMT2D, also known as MLL4, catalyzes the mono‐methylation of mammalian histone H3K4 through its catalytic function. According to previous studies, the tandem PHD domain is essential for the methyltransferase activity of KMT2D.^48^ Similarly, in our study, we found that C1523A mutation within the PHD domain disrupted the catalytic activity of KMT2D towards the histone H3K4me1 mark in TNBC cells. Moreover, we revealed YBX1 as a novel “reader” of KMT2D‐mediated H3K4me1 mark. KMT2D‐mediated H3K4me1 mark and YBX1 were colocalized at the TSS and promoter regions of the *c‐Myc* and *SENP1* genes in breast cancer cells. Interestingly, either overexpression of KMT2D^C1523A^ mutant or knockdown of KMT2D significantly reduced YBX1 binding at the TSS and promoter regions of the *c‐Myc* and *SENP1* genes, leading to decreased expression of these genes. These data together demonstrated that KMT2D‐mediated H3K4me1 recruits YBX1 to facilitate TNBC progression through epigenetic activation of c‐Myc and SENP1 expression.

Arabidopsis histone methyltransferase SDG8 was the first identified reader of H3K4me1.^52^ The crystal structure analysis revealed that SDG8 specifically binds to the H3K4me1 mark, but not H3K4me2 or H3K4me3, through its CW domain.^53^ Additionally, Local et al.^54^ reported that in mammalian cells, DPF3 within BAF complexes exhibits a preferential affinity for H3K4me1, with the PHD domain likely contributing to its selective binding towards unmethylated H3K4 or H3K4me1. We discovered YBX1 as a novel H3K4me1‐interacting protein and observed that the monomethyl group of H3K4 was enclosed within a preformed cavity, specifically designed to accommodate only one methyl group. Consequently, this structural feature blocked the binding of YBX1 to H3K4me2 or H3K4me3. Moreover, the E121A mutation in the binding pocket of YBX1 significantly impaired its binding ability to the H3K4me1 peptide, highlighting the crucial role of amino acid residue E121 of YBX1 in recognizing H3K4me1.

H3K4me1 is commonly deposited at distal enhancers accompanied by a reduction in H3K4me3. It exhibits high dynamics and shows strong correlation with cell type‐specific gene expression profiles.^15^, ^49^ However, it should be noted that H3K4me1‐enriched regions are not limited to enhancers alone. The impact of the H3K4me1 mark on promoters has been extensively investigated by multiple research groups, who have highlighted its distinct transcriptional characteristics in comparison to enhancers. For instance, Cheng et al.^55^ revealed that H3K4me1 was distributed on promoters of activated genes in embryonic fibroblasts and macrophages. These aforementioned phenomena were also observed in embryonic stem cells and germ cells.^46^ Our analysis in TNBC cells using ChIP‐seq revealed strong H3K4me1 signals at TSS, promoters, and enhancer regions, suggesting that this modification could have diverse transcriptional regulatory roles at different regulatory elements. Other reports have suggested that the varying H3K4me1/3 signal ratio at promoters may affect the recruitment of specific transcriptional factors.^46^, ^56^ Despite extensive studies on H3K4me1 at enhancers or promoters, a more precise definition of its clear function is still needed.

In cancer, c‐Myc plays an important role in cell growth, differentiation, apoptosis, and metabolism. The dysregulation of c‐Myc is observed in around 70% of human cancers, and compelling evidence suggests that aberrantly expressed c‐Myc plays a role in both tumour initiation and maintenance. Inhibiting c‐Myc has been shown to significantly reduce tumour growth in vivo, with no irreversible effects on healthy tissue. The discovery offers a valuable opportunity for cancer treatment, making c‐Myc one of the most promising targets for developing drugs. However, due to the intrinsically disordered region within its structure, c‐Myc has traditionally been considered an “undruggable” target. We revealed c‐Myc as a downstream target gene of the KMT2D‐H3K4me1‐YBX1 axis. Silencing KMT2D and YBX1 significantly inhibited c‐Myc expression in TNBC cells. Moreover, the reintroduction of c‐Myc and SENP1, two target genes of KMT2D‐H3K4me1‐YBX1 axis were identified in our study, effectively rescued cell growth and migration of KMT2D‐ or YBX1‐knockdown TNBC cells in vitro. This suggests that the KMT2D‐H3K4me1‐YBX1 axis promotes TNBC progression by regulating the expressions of c‐Myc and SENP1. However, the therapeutic potential of the KMT2D‐H3K4me1‐YBX1 axis, which relies on c‐Myc, still needs to be validated through further in vivo studies.

However, this study has limitations that warrant acknowledgement. An expanding array of small molecule inhibitors are being tested in clinical trials for their potential to combat tumours. Currently, there are no small molecule inhibitors targeting KMT2D and YBX1 available on the market. Our in vivo study only demonstrated that gene silencing of KMT2D and YBX1 inhibits growth and lung metastasis in TNBC. We are also considering the development of inhibitors targeting the enzymatic activity of KMT2D or small molecule inhibitors to block the binding of YBX1 to H3K4me1 for the treatment of TNBC. Therefore, further studies will consolidate our conclusions.

In summary, we characterized YBX1 as an H3K4me1‐binding protein in TNBC cells. KMT2D acts cooperatively with YBX1 to facilitate TNBC progression through epigenetic activation of c‐Myc and SENP1 transcription. These findings unveil a crucial interaction between histone marks and transcriptional modulation during the progression of TNBC, thereby supporting strategies targeting the KMT2D‐H3K4me1‐YBX1 axis to treat breast cancer.

## AUTHOR CONTRIBUTIONS

Bing Yao,Quan Zhao and Changyan Ma designed the experiments and prepared the manuscript. Bing Yao, Mengying Xing and Xiangwei Zeng performed most experiments. Ming Zhang, Zhi Wang, and Bo Peng contributed to the computational statistical analysis, which was supervised by Changyan Ma and Haitao Li. Que Zheng, Shuang Qu, Lingyun Li, Yucui Jin and Hongyan Yuan performed a specific subset of the experiments and analyses. All authors have read and approved the final manuscript.

## CONFLICT OF INTEREST STATEMENT

The authors declare no conflict of interest.

## ETHICS STATEMENT

Written informed consent was obtained from all patients and the study was approved by the Ethics Committee of Shang Hai Outdo Biotech Co, Ltd (SHYJS‐CP‐1607001; SHYJS‐CP‐1607006). All animal experimental procedures were conducted in accordance with animal protocols approved by the Laboratory Animal Center of Nanjing Medical University (IACUC‐2011053).

## Supporting information

Supporting information

## Data Availability

The datasets used in the current study are available from the corresponding author upon reasonable request.
