# Supplementary material for: KMT2D‐mediated H3K4me1 recruits YBX1 to facilitate triple‐negative breast cancer progression through epigenetic activation of c‐Myc
Source: Clin Transl Med. 2024 Jul 5;14(7):e1753. doi: 10.1002/ctm2.1753 (PMC11225074; doi:10.1002/ctm2.1753)
Supplement: Supplementary file 1 — Supporting information [file CTM2-14-e1753-s001.docx]

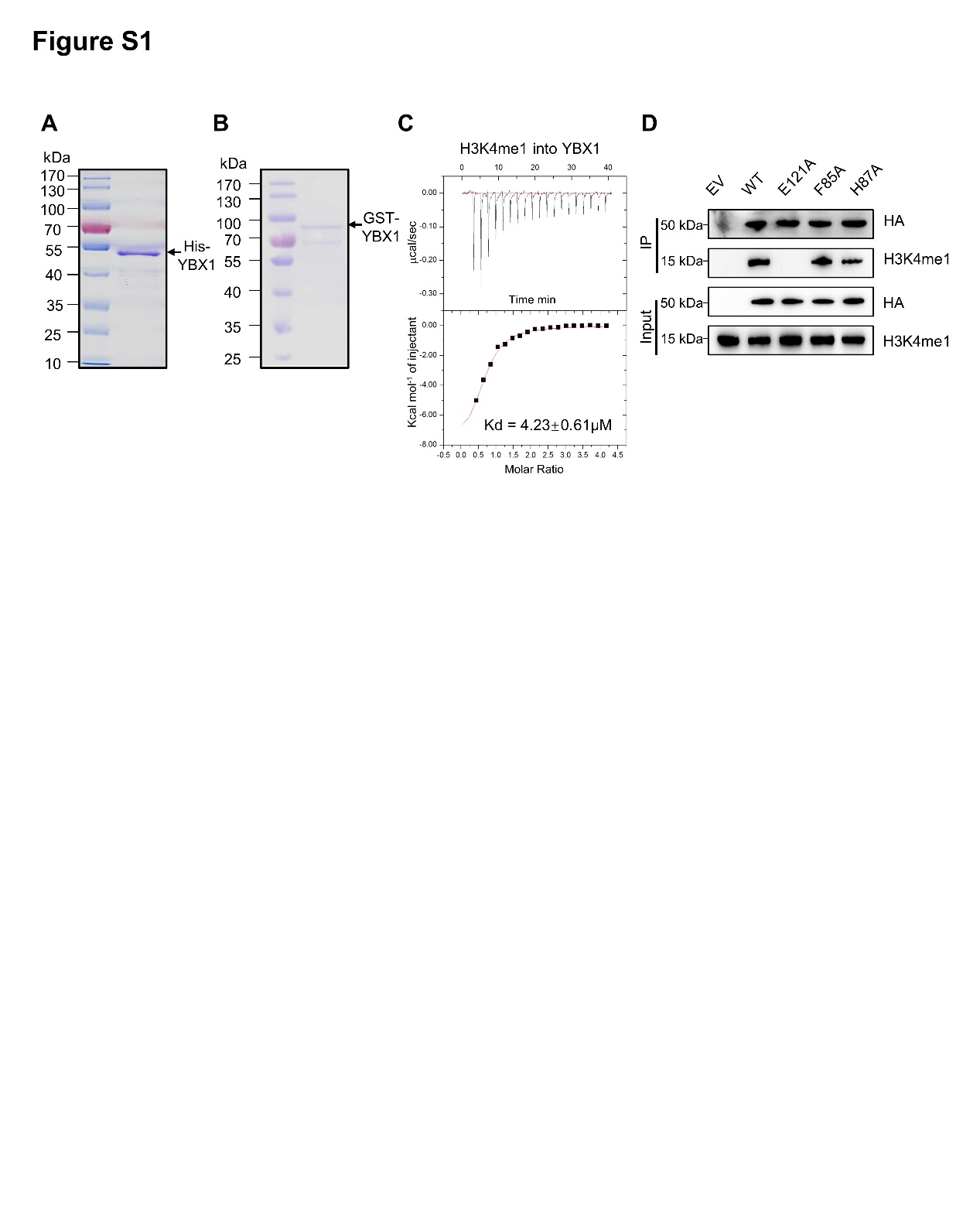


**Figure S1. YBX1 is an H3K4me1-binding protein and YBX1-E121 is necessary for its binding.** (A-B) Coomassie blue staining of purified His-YBX1 (A) and GST-YBX1 (B). (C) ITC binding curves of YBX1 recombinant protein with H3K4me1 peptides. Kd = 4.23 ± 0.61 μM. (D) Co-IP assay of proteins immunoprecipitated with HA antibodies from lysates of MDA-MB-231 cells transfected with empty vector (EV), HA-YBX1-WT, HA-YBX1-E121A, HA-YBX1-F85A and HA-YBX1-H87A.


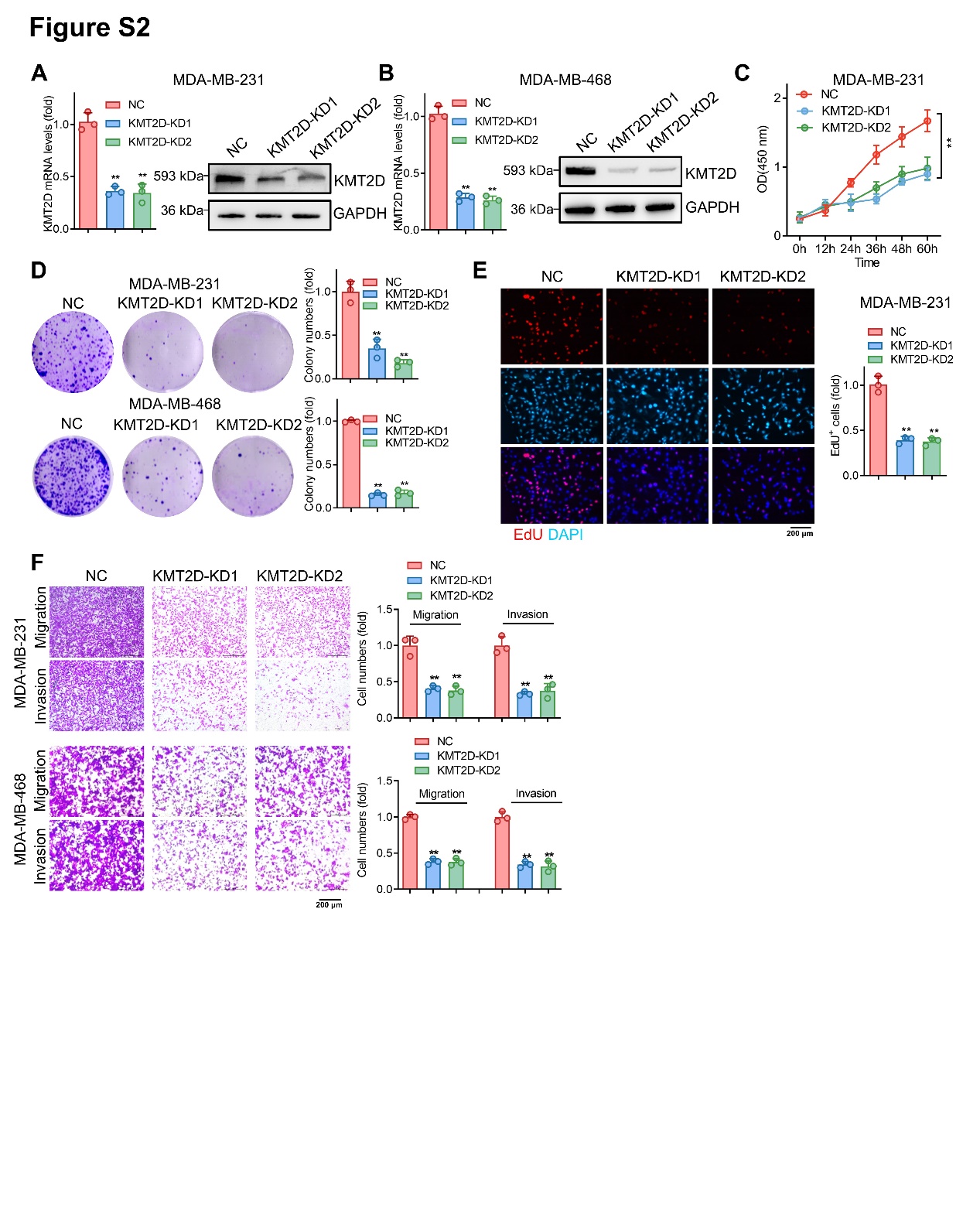


**Figure S2. KMT2D and YBX1 promote TNBC cell proliferation and migration *in vitro*.** (A-B) qRT-PCR and western blot analyses of KMT2D mRNA and protein levels in MDA-MB-231 (A) and MDA-MB-468 (B) cells transfected with negative control (NC) or KMT2D siRNAs. (C) CCK-8 assay to determine the proliferation of NC and KMT2D-knockdown (KMT2D-KD) MDA-MB-231 cells. (D) Plate colony-formation assay to determine the proliferation of NC and KMT2D-KD MDA-MB-231 and MDA-MB-468 cells. (E) EdU incorporation assay to determine the proliferation of NC and KMT2D-KD MDA-MB-231 cells. (F) Migration and invasion assay to determine the migration and invasion of NC and KMT2D-KD MDA-MB-231 and MDA-MB-468 cells.


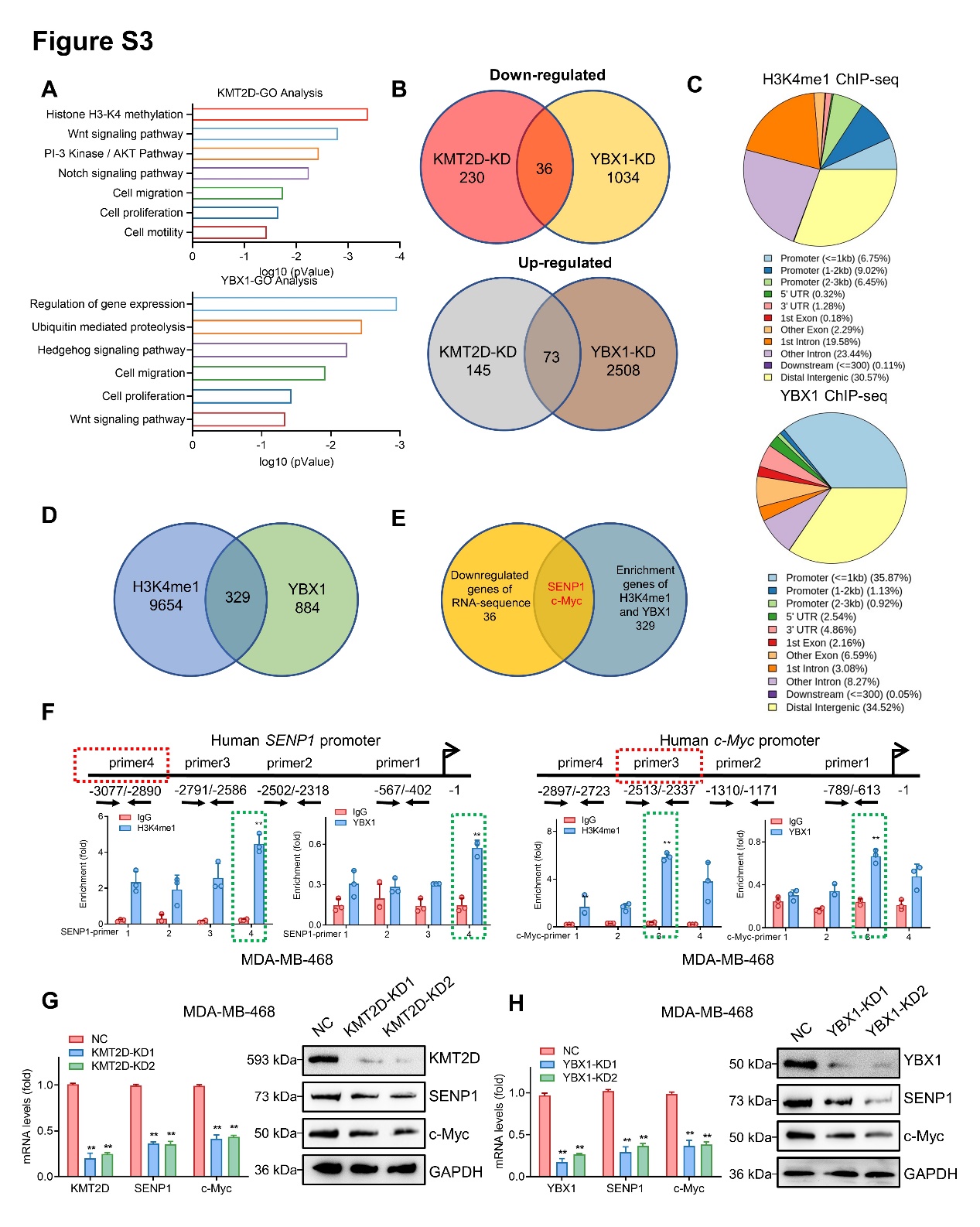


**Figure S3. c-Myc and SENP1 are target genes of KMT2D and YBX1 in TNBC cells.** (A) Gene Ontology (GO) functional analysis for the differentially expressed genes (DEGs) in MDA-MB-231 cells following KMT2D or YBX1 knockdown. (B) Venn diagrams showing overlapping of differentially expressed genes (DEGs) in MDA-MB-231 cells following KMT2D and YBX1 knockdown. (C) Peak distribution of H3K4me1 mark and YBX1 in the genome from our ChIP-seq data. (D) Venn diagram showing overlapping genes of H3K4me1 and YBX1 identified by ChIP-seq analysis in MDA-MB-231 cells. (E) Venn diagram showing the overlapping genes of KMT2D and YBX1 identified by RNA-seq and ChIP-seq analyses in MDA-MB-231 cells. (F) ChIP assay showing the binding of H3K4me1 and YBX1 to c-Myc and SENP1 promoters in MDA-MB-468 cells. (G) qRT-PCR and western blot analyses of c-Myc and SENP1 mRNA and protein levels in NC and KMT2D-KD MDA-MB-468 cells. (H) qRT-PCR and western blot analyses of c-Myc and SENP1 mRNA and protein levels in NC and YBX1-KD MDA-MB-468 cells.


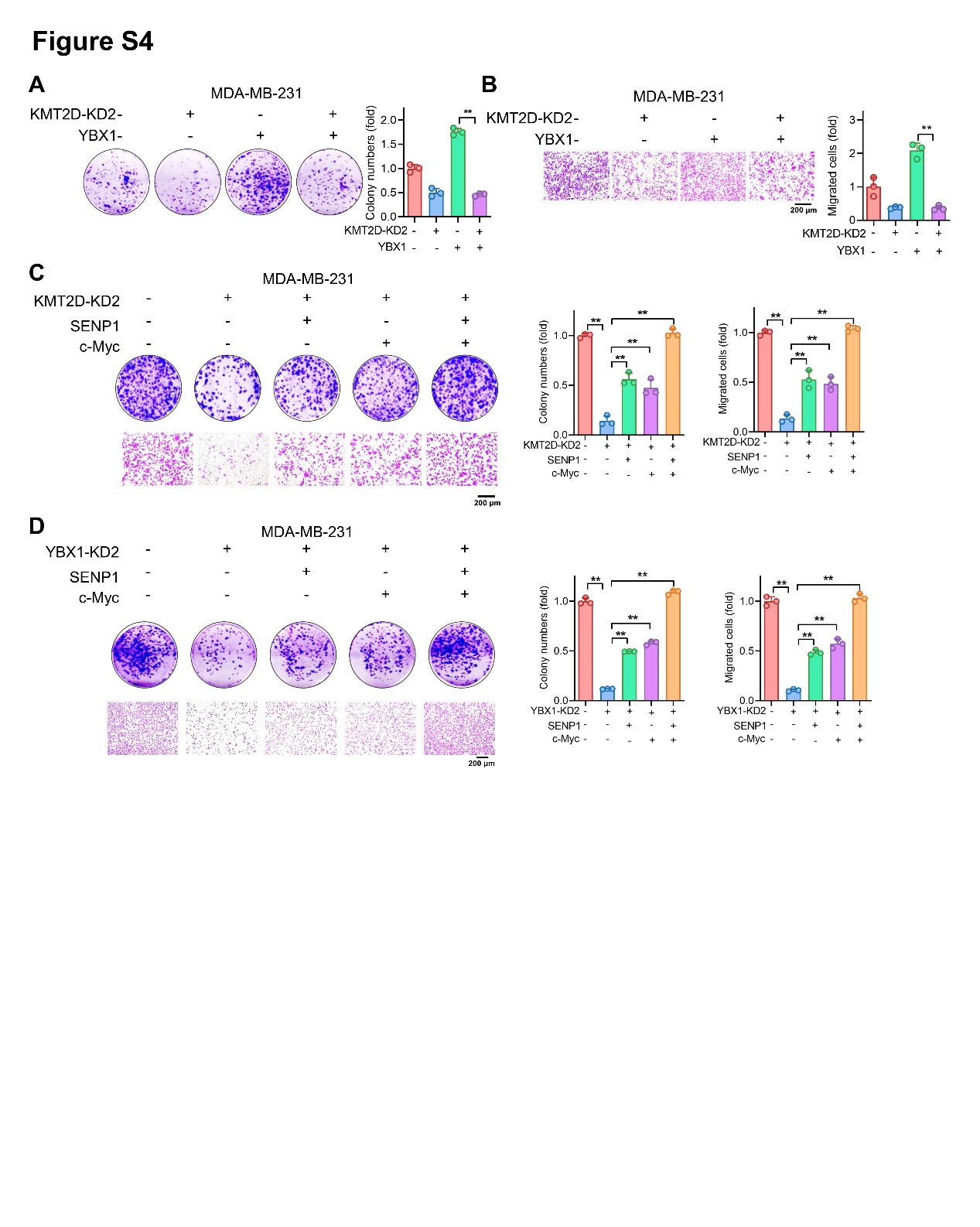


**Figure S4. KMT2D and YBX1 cooperatively promote TNBC cell proliferation and migration *in vitro.*** (A) Colony-formation assays of MDA-MB-231 cells transfected with NC, KMT2D-KD2, YBX1, KMT2D-KD2 + YBX1. (B) Migration assays of MDA-MB-231 cells transfected with NC, KMT2D-KD2, YBX1, KMT2D-KD2 + YBX1. (C) Colony-formation and migration assays of MDA-MB-231 cells transfected with NC, KMT2D-KD2, SENP1 or c-Myc. Representative images (left panels) and quantitative analyses of the colonies or migrated cells (right panels) are shown. (D) Colony-formation and migration assays of MDA-MB-231 cells transfected with NC, YBX1-KD2, SENP1 or c-Myc. Representative images (left panels) and quantitative analyses of the colonies or migrated cells (right panels) are shown. Data were showed as mean ± SD. **P* < 0.05, ***P* < 0.01.


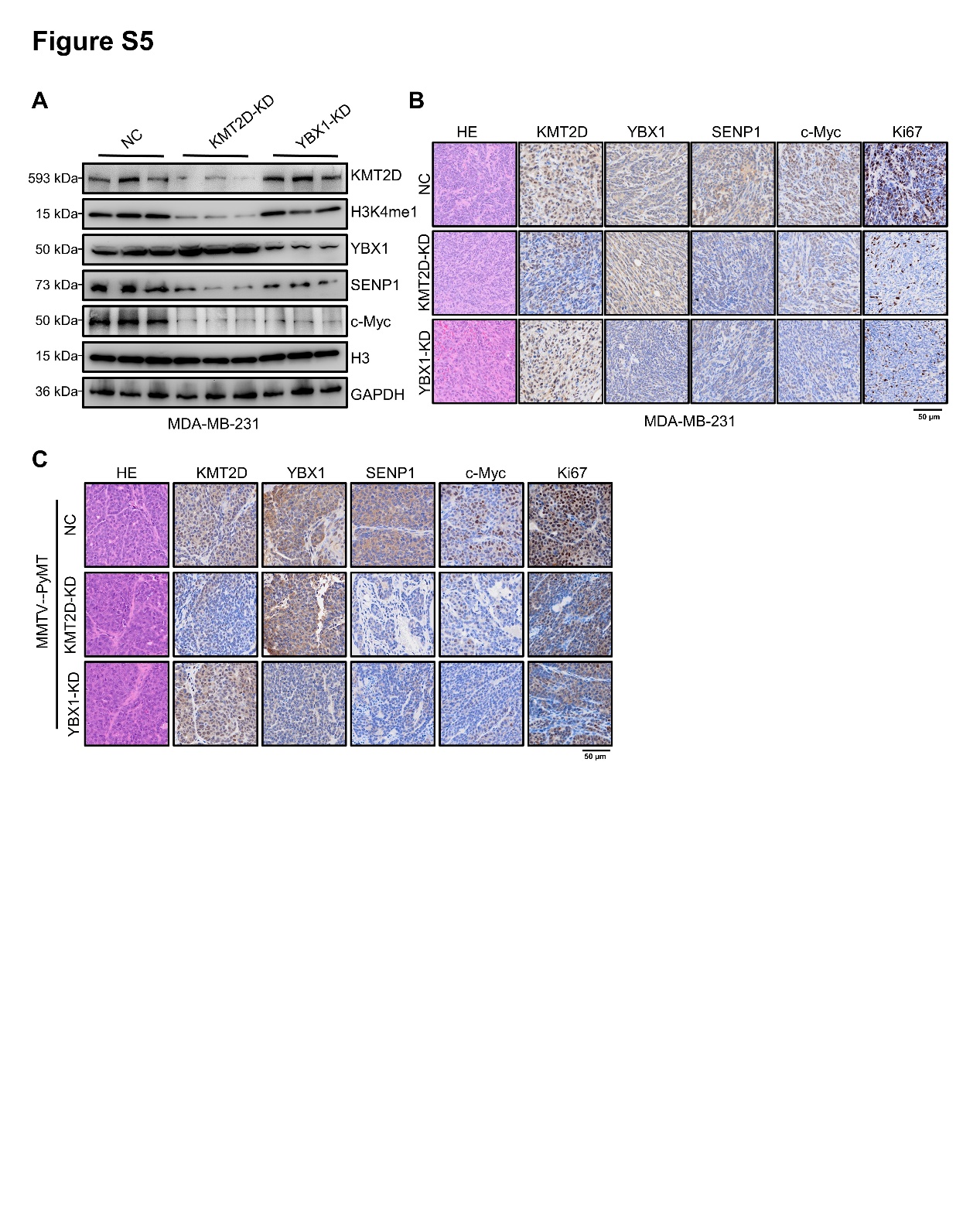


**Figure S5. Knockdown of KMT2D or YBX1 attenuates breast tumor growth and metastasis *in vivo.*** (A) Western blot analyses of c-Myc and SENP1 protein levels in KMT2D-knockdown (KMT2D-KD) or YBX1-knockdown (YBX1-KD) MDA-MB-231 xenografts. (B) Representative H&E and IHC staining (KMT2D, YBX1, SENP1, c-Myc and Ki-67) images of NC, KMT2D-knockdown (KMT2D-KD) and YBX1-knockdown (YBX1-KD) MDA-MB-231 xenografts. Scale bar = 50 μm. (C) Representative H&E and IHC staining (KMT2D, YBX1, SENP1, c-Myc and Ki-67) images of mammary tumors from NC, KMT2D-KD and YBX1-KD AAV-treated MMTV-PyMT transgenic mice. Scale bar = 50 μm.


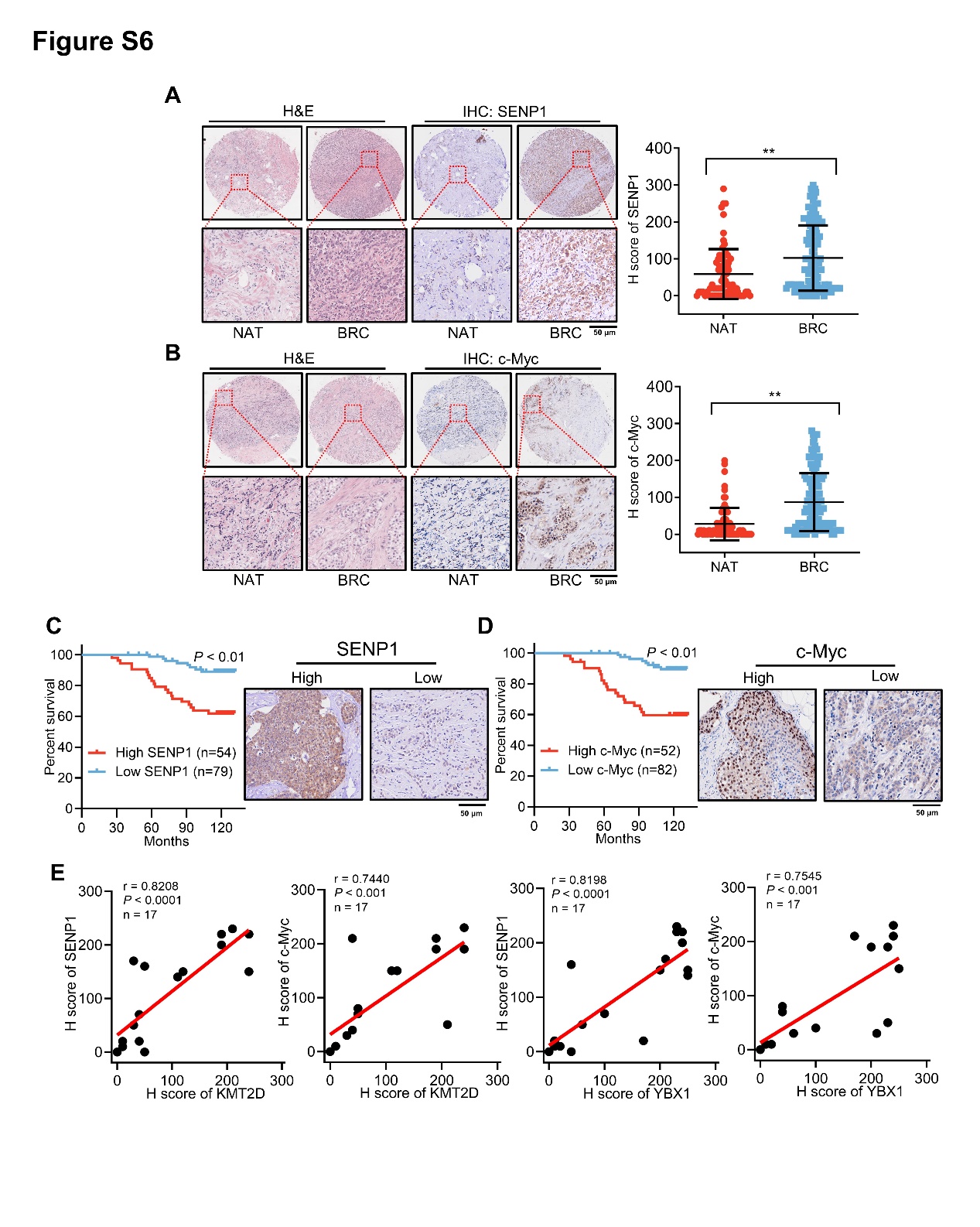


**Figure S6. Elevated KMT2D and YBX1 expression positively correlates with c-Myc and SENP1 expression and predicts a poor prognosis in breast cancer patients.** (A) H&E and IHC staining of SENP1 protein in adjacent normal (NAT) and breast cancer (BRC) tissues. Scale bar = 50 μm. Right panel shows the IHC score of SENP1 in NAT and BRC tissues. (B) H&E and IHC staining of c-Myc protein in NAT and BRC tissues. Scale bar = 50 μm. Right panel shows the IHC score of c-Myc in NAT and BRC tissues. (C) Kaplan-Meier plot of overall survival of 133 patients with breast cancer, stratified by SENP1 expression (Log-rank test, *P* < 0.01). (D) Kaplan-Meier plot of overall survival of 134 patients with breast cancer, stratified by c-Myc expression (Log-rank test, *P* < 0.01). (E) Pearson correlation scatter plots of H scores of SENP1 and KMT2D (r = 0.8208, *P* < 0.0001), c-Myc and KMT2D (r = 0.7440, *P* < 0.001), SENP1 and YBX1 (r = 0.8198, *P* < 0.0001), c-Myc and YBX1 (r = 0.7545, *P* < 0.001) in TNBC tissues.

**Table S1 List of proteins identified by Mass Spectrometry.**

**Table S2 Clinicopathologic characteristics of KMT2D and YBX1 expression in BRC patients.**

| **Characteristics** | **Cases** | **IHC score of KMT2D** | |  | **IHC score of YBX1** | |
| --- | --- | --- | --- | --- | --- | --- |
|  |  | **Mean±s.d.** | ***P* value^a^** |  | **Mean±s.d.** | ***P* value^a^** |
| Age  ＞50  ≤50 | 89  51 | 107.2 ± 8.7  101.3 ± 11.5 | 0.6900 |  | 114.8 ± 8.3  115.0 ± 12.9 | 0.9907 |
| Grade  *II*  *II-III, III* | 98  42 | 97.5 ± 8.4  122.4 ± 12.1 | 0.0979 |  | 100.3 ± 8.1  147.6 ± 12.2 | 0.0016 |
| Tumor Size  *T1*  *T2-T3* | 57  83 | 83.8 ± 8.6  120.0 ± 9.8 | 0.0097 |  | 87.4 ± 10.5  133.7 ± 8.8 | 0.0010 |
| Lymph node status  *N_0_*  *N_1-3_* | 75  65 | 97.8 ± 9.5  113.9 ± 10.1 | 0.2482 |  | 108.2 ± 9.9  122.8 ± 9.9 | 0.3007 |
| *Recurrence*  *Yes*  *No* | 38  102 | 141.8 ± 13.2  92.7 ± 7.8 | 0.0018 |  | 140.6 ± 13.2  106.1 ± 8.1 | 0.0309 |

^a^ *P* values were derived using Student’s *t*-test to compare values for the two parameters in each category.

**Table S3 36 common genes down-regulated by KMT2D-knockdown and YBX1-knockdown in MDA-MB 231 cells.**

**Table S4 329 common genes of H3K4me1 and YBX1 identified by ChIP-seq analysis in MDA-MB 231 cells.**

**Table S5 Antibodies.**

| **Antibodies** | **Source** | **Identifier** |
| --- | --- | --- |
| Anti-YBX1  Anti-KMT2D  Anti-KMT2D  Anti-H3K4me1  Anti-H3K4me1  Anti-H3K4me3  Anti-H3  Anti-His  Anti-HA  Anti-GAPDH  Anti-Flag  Anti-SENP1  Anti-c-Myc  Anti-Ki67 | Abcam  Affinity  Abcam  Abcam  Active motif  Abcam  Abcam  Yifeixue  CST  Abcam  CST  Abcam  Abcam  Abcam | ab239875  DF9646  ab224156  ab176877  39635  ab213224  Ab1791  YFMA0041  #3724  ab8245  #14793  ab108981  ab32072  ab15580 |

**Table S6 Peptides.**

| **Peptides, chemicals** | **Source** | **Identifier** |
| --- | --- | --- |
| H3  H3K4me1  H3K4me3 | Genscript, Nanjing, China  Genscript, Nanjing, China  Genscript, Nanjing, China | C438XEJ300  C438XEJ301  C438XEJ302 |

**Table S7 Primers for RT-PCR.**

| **Names** | **Sequence (5’-3’)** |
| --- | --- |
| YBX1-F  YBX1-R  KMT2D-F  KMT2D-R  SENP1-F  SENP1-R  c-Myc-F  c-Myc-R  GAPDH-F  GAPDH-R | GGGGACAAGAAGGTCATCGC  CGAAGGTACTTCCTGGGGTTA  GAGCTACGGCGCTTTGAGTT  AGGGAAACCAATCTGTGATAGGT  CTTGGCTCAGGCGATTTAAGA  GAGGTAAAGACTTCGGCTGTT  GTCAAGAGGCGAACACACAAC  TTGGACGGACAGGATGTATGC  GGAGCGAGATCCCTCCAAAAT  GGCTGTTGTCATACTTCTCATGG |

**Table S8 Primers for ChIP.**

| **Names** | **Sequence (5’-3’)** |
| --- | --- |
| SENP1-ChIP1-F  SENP1-ChIP1 -R  SENP1-ChIP2-F  SENP1-ChIP2-R  SENP1-ChIP3-F  SENP1-ChIP3-R  SENP1-ChIP4-F  SENP1-ChIP4-R  C-Myc-ChIP1-F  C-Myc-ChIP1-R  C-Myc-ChIP2-F  C-Myc-ChIP2-R  C-Myc-ChIP3-F  C-Myc-ChIP3-R  C-Myc-ChIP4-F  C-Myc-ChIP4-R | CTTGTTTCCTAGTCCTAGA  CTGAGATCTAAATCGATCTC  CTCATTTAGTCAACTTTCT  AGAGAGGTGGGGATGGATC  GTGTAAGCTATTGGGGCA  TAAATGTTCAATTCACAGGC  GTTCTTAACTTACACATTG  AAGTAACAACTCCTAGCAC  CTGAGCAGGCGGGGCAGGA  CCACGTATACTTGGAGAG  GGCACGGAAGTAATACTC  ACTCTTTCCTCCCCGGAC  GTGACAGAAGGAGACCCTG  CTTTAGCCCCCACGTCTTAG  GCTCCTATTCCTTCACAC  CTTCGGCCTTTGCCTCTG |

**Table S9 Viruses.**

| **Name** | **Source** |
| --- | --- |
| Mouse-AAV-KMT2D-RNAi  Mouse-AAV-YBX1-RNAi  Mouse-AAV-Control-RNAi | Beijing Synthetic Biological Technology  Beijing Synthetic Biological Technology  Beijing Synthetic Biological Technology |

**Table S10 Tissue Arrays.**

| **Name** | **Source** |
| --- | --- |
| HBreD140sU06 (140 BRC tumor tissues from patient)  HBreD077Su01 (77 BRC adjacent tissues from patient) | Shang Hai Outdo Biotech Co, Ltd.  Shang Hai Outdo Biotech Co, Ltd. |
